# Astrocyte-secreted chordin-like 1 regulates spine density after ischemic injury

**DOI:** 10.1038/s41598-022-08031-4

**Published:** 2022-03-09

**Authors:** Elena Blanco-Suarez, Nicola J. Allen

**Affiliations:** 1 Molecular Neurobiology Laboratory, Salk Institute for Biological Studies, 10010 N Torrey Pines Road, La Jolla, CA 92037 USA; 2 Present Address: Department of Neuroscience, Thomas Jefferson University, 900 Walnut Street, Philadelphia, PA 19107 USA

**Keywords:** Astrocyte, Stroke

## Abstract

Ischemic injury occurs when the brain is deprived of blood flow, preventing cells from receiving essential nutrients. The injury core is the brain region directly deprived and is surrounded by the peri-infarct area, the region with recovery potential. In the peri-infarct area neurons undergo acute loss of dendritic spines, which modifies synaptic plasticity and determines neuronal survival. Astrocytes can be protective or detrimental to the ischemic injury response depending on the specific stage, yet we lack clear understanding of the underlying mechanisms. Chordin-like 1 (Chrdl1) is an astrocyte-secreted protein that promotes synaptic maturation and limits experience-dependent plasticity in the mouse visual cortex. Given this plasticity-limiting function we asked if Chrdl1 regulates the response to ischemic injury, modelled using photothrombosis (PT). We find that Chrdl1 mRNA is upregulated in astrocytes in the peri-infarct area in both acute and sub-acute phases post-PT. To determine the impact of increased Chrdl1 on the response to PT we analyzed Chrdl1 knock-out mice. We find that absence of Chrdl1 prevents ischemia-induced spine loss in the peri-infarct area and reduces cell death in the core, without impacting gliosis. These findings highlight the important role of astrocyte-secreted proteins in regulating structural plasticity in response to brain ischemic injuries.

## Introduction

Ischemic injury is characterized by the interruption of blood flow to a specific region of the brain. This interruption causes an acute depletion of glucose and oxygen, preventing cellular metabolic function, leading to cell death and tissue damage, and eventual loss of brain function^1^. The brain region depleted of blood flow is referred to as the core of the injury. In response to ischemic insults, there is an excessive release of glutamate that over-activates glutamatergic receptors, a process known as excitotoxicity that ultimately leads to neurodegeneration^2^. Mostly, the cells confined to the core of the injury follow apoptotic and necrotic pathways causing cell death and preventing neuronal rescue. The surrounding tissue suffers from a decreased blood supply, and it is called the peri-infarct area. In this region, diverse cellular and molecular mechanisms are triggered in response to the reduced blood supply and cell metabolism, which will dictate whether cells in the peri-infarct area survive or follow delayed cell death^3^. It is the peri-infarct area that holds potential for recovery, and understanding the molecular mechanisms that take place in this region post-injury is crucial to promote cell survival over delayed cell death.

Several methods are available to mimic brain ischemia in mouse models^4^. In the present study we use photothrombosis (PT), a technique that consists of transcranial illumination of a brain region upon injection with Rose Bengal, a photoactivatable dye. This induces the formation of singlet oxygen that damages the endothelium, promoting platelet aggregation and formation of thrombi that block the blood supply to the illuminated area, causing similar effects to focal ischemia in humans^5^. After ischemic injury, there are different phases characterized by the ability for spontaneous recovery in the peri-infarct area. In the mouse, these phases are divided into acute (0–2 days post-injury), sub-acute (2–30 days post-injury) and chronic (beyond 30 days post-injury)^6^, with comparable time windows for humans^7^. At the beginning of the acute phase there is a high rate of cell death in combination with the onset of inflammatory mechanisms. At the end of the acute phase, the formation of an astrocytic scar begins and extends into the following sub-acute phase^8^. The sub-acute phase is the time window when endogenous plasticity mechanisms take place to support neural repair. The level of repair displayed during the chronic phase is determined during the sub-acute phase as it is the time when a certain degree of tissue reorganization, including the further development of the astrocytic scar, takes place. At the late chronic stage, endogenous mechanisms of plasticity are diminished and neural repair is minimal^6^. Currently, it is unclear what triggers each phase post-injury, and how to harness the potential of endogenous plasticity mechanisms to promote functional recovery and neural repair at later stages (i.e., chronic phase). There is evidence that during the sub-acute phase there is increased expression of genes related to synaptogenesis and spinogenesis^9^, which enhances endogenous plasticity and spontaneous recovery. Thus, the study of structural plasticity in the context of ischemia holds great interest in order to understand the mechanisms that make post-stroke plasticity and spontaneous recovery the most efficient to achieve functional recovery.

Astrocytes play important roles in homeostasis of the central nervous system (CNS), and some of these functions are of particular importance in the context of ischemic injuries, pointing to astrocytes as potential targets for neuroprotection^10^. Astrocytes are important components of the neurovascular unit contributing to the integrity of the blood–brain barrier (BBB); they play an important role in glial scar formation; and they are responsible for clearing excessive glutamate from the extracellular space which otherwise triggers excitotoxic events^11^. In addition, some evidence has shown how astrocytes and microglia may be involved in diverse CNS disorders and pathologies, including ischemic stroke^12,13^. Astrocytes, in response to injury and disease, go through changes in their morphology, molecular profile and functionality, a response termed astrogliosis, regulated by signaling pathways that are yet to be fully defined^14^. Astrocytes are not isolated, and in fact interact with a variety of cells that surround the peri-infarct area to regulate different responses such as inflammation or tissue replacement^8^. In response to stroke, a range of inflammatory cells, such as neutrophils, macrophages, T cells and microglia, get activated and infiltrate the injury site, promoting both detrimental and beneficial effects^15^. Microglia are crucial in the regulation of the immune response in the CNS during acute injuries like stroke. They undergo morphological changes and they are recruited to the injury site where they release a series of proinflammatory molecules with potential injurious effects at early stages after injury^16^. At chronic post-injury stages, microglia release anti-inflammatory and neurotrophic factors with neuroprotective functions^16^. Both astrocytes and microglia partake in inflammatory mechanisms during ischemia, which promotes both detrimental and beneficial effects^15^.

In the healthy brain astrocytes modulate the development, maturation and function of synapses through the secretion of diverse factors^17^, and some of these factors have been linked to neuroprotective mechanisms in ischemic injuries. For example, thrombospondins-1 and -2 (TSP-1 and -2) are astrocyte-secreted proteins involved in the formation of silent synapses^18^. Elimination of TSP-1 and -2 leads to a greater loss of synapses in response to ischemic lesions which impairs functional recovery, therefore establishing an important role for astrocyte-secreted proteins in synaptic regulation after stroke^19^. We previously identified chordin-like 1 (Chrdl1), which is enriched in expression in astrocytes in upper layers of the cortex and the striatum, as an astrocyte-secreted protein that induces synapse maturation by recruiting GluA2 AMPA glutamate receptors to synaptic sites^20^. During normal development the visual critical period is a time when endogenous plasticity is enhanced in the visual cortex, permitting remodeling of visual circuits in response to a modification in visual input^21^. This endogenous plasticity is greatly diminished at older ages. We demonstrated that the absence of Chrdl1 increases experience-dependent plasticity in a visual sensory deprivation paradigm, an effect that was observed not only during periods of high endogenous plasticity i.e. the visual critical period, but beyond this time into adulthood, identifying Chrdl1 as an important negative regulator of synaptic plasticity^20^. A study blocking a different plasticity-limiting molecule that is expressed by neurons, PirB, found this manipulation also promoted enhanced experience-dependent plasticity, and further proved beneficial in the context of ischemic stroke by reducing the size of the injury, improving motor recovery, and decreasing astrocyte reactivity^22,23^. This suggests that targeting other endogenous plasticity-limiting molecules, such as Chrdl1, may be beneficial in recovery from ischemic injuries.

To enable functional recovery after ischemia, plasticity potential is necessary to promote functional and structural changes to compensate for lost synapses^24^. As Chrdl1 regulates synaptic maturation and limits plasticity, we asked what role Chrdl1 plays in regulating the neuronal response to PT-injury. We found that Chrdl1 is significantly upregulated during the acute phase (24 h post-injury) in both the peri-infarct and contralateral hemispheres, whereas at later timepoints during the sub-acute phase (7 days post-injury) the expression of Chrdl1 remains elevated only in the peri-infarct area, and returns to physiological levels during the chronic phase (30 days post-injury). Elimination of Chrdl1 in vivo reduces cell death and prevents the characteristic spine loss associated with ischemic injuries that has been previously reported^25^. Therefore, we hypothesize that the increase in plasticity-potential in the absence of the astrocyte-secreted protein Chrdl1 reduces cell death, permits faster remodeling in the peri-infarct area and potentially promotes functional recovery.

## Results

### Chrdl1 expression increases in astrocytes in response to ischemic conditions

In response to ischemia caused by MCAO (middle cerebral artery occlusion), the expression of various astrocytic genes is altered^26^. For example, SPARC and TSP-1, both astrocyte-secreted factors with synaptogenic roles, are upregulated during the acute phase and they have been linked to functional recovery^19,27^. Here we used photothrombosis (PT) to cause ischemic injury in the visual cortex of 4-month-old male mice to study the role of Chrdl1. The effects were analyzed during the acute (24 h post-injury), sub-acute (7 days post-injury) and chronic (30 days post-injury) phases, the post-injury stages characterized by different plasticity levels. The sub-acute phase is considered the time window during which, given the enhanced endogenous plasticity, recovery and neural repair are facilitated^7^.

**Figure 1:**
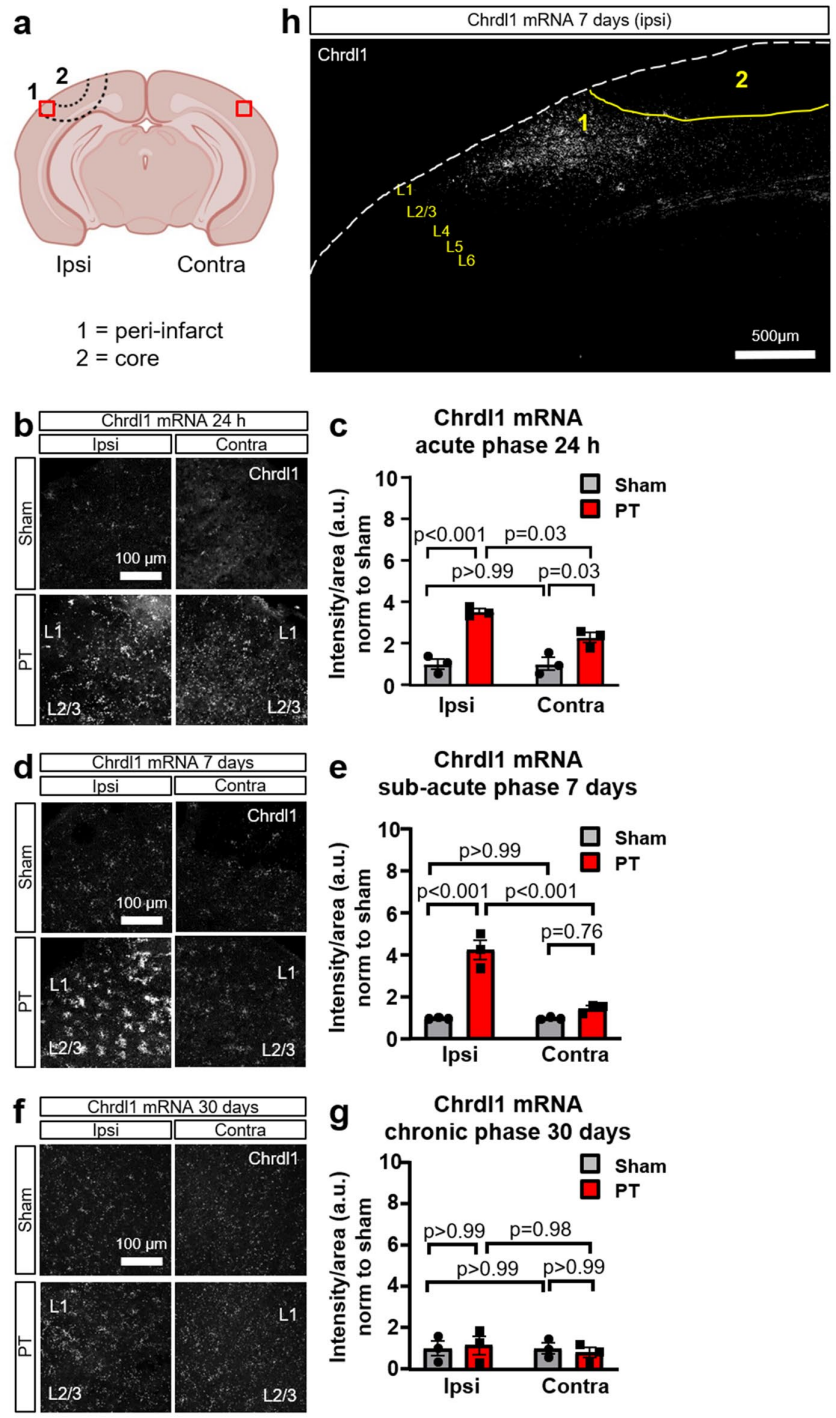
Chrdl1 expression increases in astrocytes in response to ischemic conditions. (**a**) Schematic drawing of a coronal section of a mouse brain to illustrate where the ischemic injury was applied. Region labelled as 1 delimited by two dashed lines indicates the peri-infarct area, and label 2 delimited by a dashed line corresponds to the core, the brain region illuminated with the laser in order to cause the photothrombotic lesion. Red boxes approximately indicate the region of interest (ROI) imaged and analyzed in all experiments in the hemisphere of the injury (ipsi) and the homologous contralateral region (contra), spanning 200 µm from the border of the core, unless noted differently. (**b**) Representative images of fluorescent in situ hybridization (FISH) of Chrdl1 mRNA in the peri-infarct area (ipsilateral hemisphere, ipsi) and in the homologous contralateral hemisphere (contra) to the ischemic lesion of coronal sections 24 h after PT or sham surgeries in WT mice. Images were taken on layers 2/3 of the visual cortex. (**c**) Quantification of fluorescence intensity per unit area, normalized to the sham condition. Sham N = 3, PT N = 3 mice. (**d**,**e**) Same as (**b**,**c**) 7 days after PT or sham surgeries. Sham N = 3, PT N = 3 mice. (**f**,**g**) Same as (**b**,**c**) 30 days after PT or sham surgeries. Sham N = 3, PT N = 3 mice. Statistics by two-way ANOVA. Scale bar 100 µm. (**h**) Overview of the visual cortex of a WT mouse 7 days after PT showing that upregulation of Chrdl1 mainly occurs in the upper layers of the peri-infarct area (labelled as 1). Scale bar 500 µm.

Due to its role in synaptic plasticity, and previous microarray data showing that the expression of different astrocytic genes is altered following MCAO^26^, we hypothesized that Chrdl1 expression could be regulated in response to ischemia in confined areas of the injured brain. By fluorescence in situ hybridization (FISH), we analyzed mRNA expression of Chrdl1 in the peri-infarct area (ipsilateral, ipsi) and in the homologous contralateral (contra) hemisphere (Fig. 1a). We found that expression of Chrdl1, which we previously demonstrated to be astrocyte-enriched^20^, was upregulated in the peri-infarct area of WT mice during the acute phase (24 h after insult) (Fig. 1b,c, ipsi: PT, 3.54 ± 0.13-fold compared to sham). This increase in the expression of Chrdl1 was also observed in non-injured tissue on the contralateral hemisphere to the lesion (Fig. 1b,c, contra: PT, 2.25 ± 0.24-fold compared to sham). During the sub-acute phase (7 days post-injury) the expression of Chrdl1 in the contralateral hemisphere was restored to physiological levels (Fig. 1d,e contra: PT, 1.47 ± 0.12-fold compared to sham), whereas the increased expression in the peri-infarct area was persistent and significantly increased (Fig. 1d,e ipsi: PT, 4.24 ± 0.47-fold compared to sham). During the chronic phase (30 days post-injury) expression of Chrdl1 returned to physiological levels in both ipsilateral and contralateral hemispheres (Fig. 1f,g, ipsi: PT, 1.11 ± 0.42-fold compared to sham, contra: PT, 0.76 ± 0.23-fold compared to sham). We found that upregulation of Chrdl1 was confined to the peri-infarct area, and mainly in the upper layers of the visual cortex, the region that shows highest expression of Chrdl1 in the healthy brain (7 days post-injury, sub-acute phase Fig. 1h).

**Figure 2:**
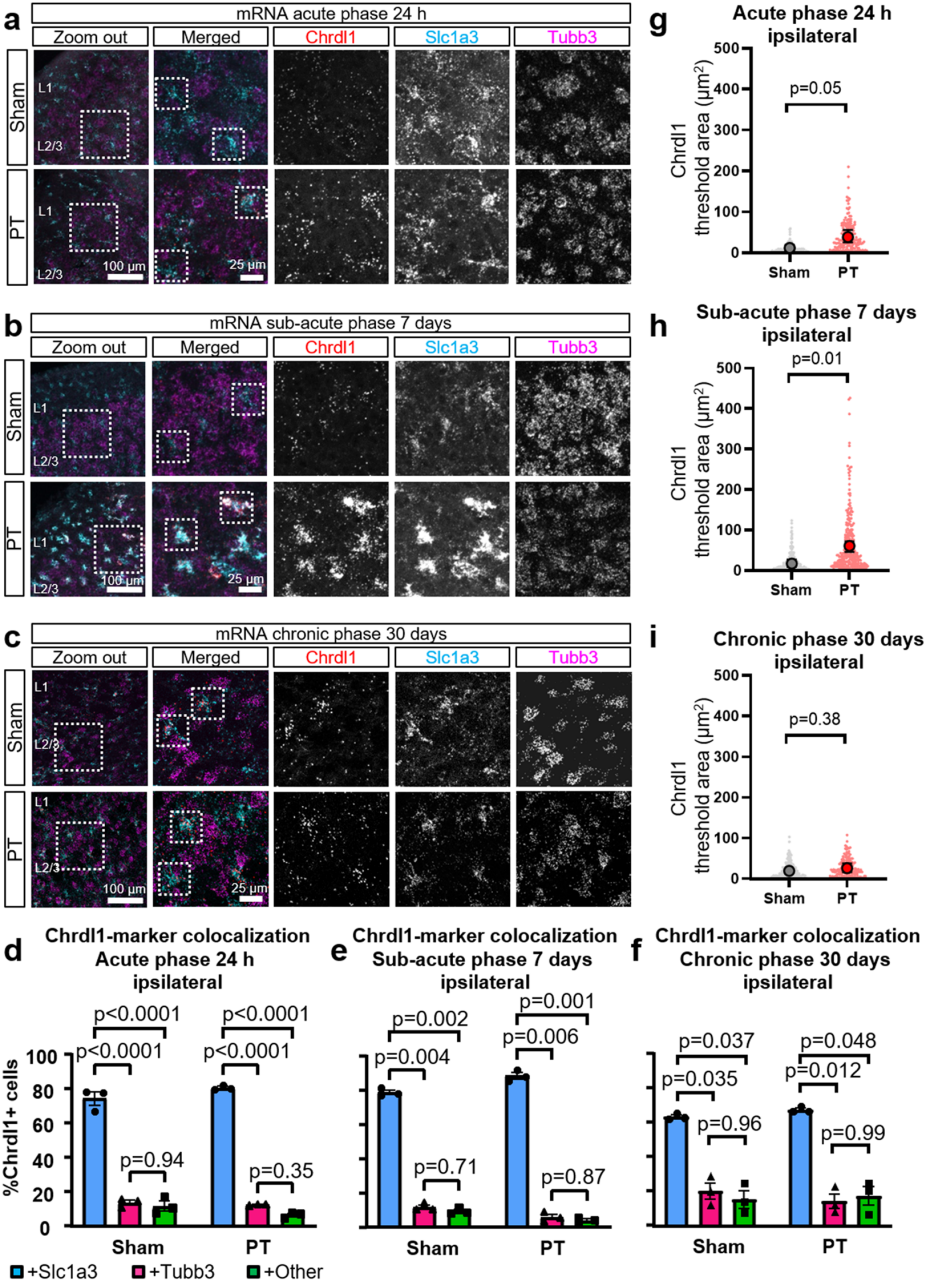
Astrocytes are the main cells expressing Chrdl1 in the peri-infarct area. (**a**) Representative images of coronal sections of the peri-infarct area in the visual cortex of WT mice 24 h after PT or sham surgeries. The zoom-out image indicates the ROI that was analyzed (scale bar 100 µm), and the dashed box shows the zoomed-in layers 2/3. FISH of Chrdl1 (in red), Slc1a3 (GLAST, in cyan), and Tubb3 (in magenta) in the peri-infarct area (ipsilateral hemisphere). Boxes in the merged image (scale bar 25 µm), indicate examples of astrocytes showing overlapping of Slc1a3 and Chrdl1. (**b**,**c**) Same as (**a**), but in WT mice 7 days and 30 days after PT or sham surgeries, respectively. (**d**) Quantification at 24 h after PT of overlapping signal between Chrdl1 and Slc1a3 (astrocyte marker), or Chrdl1 and Tubb3 (neuronal marker), or Chrdl1 and other cells. Sham N = 3, PT N = 3 mice. (**e**,**f**) Same as (**c**) but 7 days and 30 days after PT or sham surgeries, respectively. Sham N = 3, PT N = 3 mice both timepoints. Statistics by two-way ANOVA. (**g**–**i**) Scatter plots represent quantification of threshold area of Chrdl1 in single astrocytes at each time point (24 h, 7 days, and 30 days after PT). Mean ± range is represented. Sham N = 3, PT N = 3 mice per time point. Statistics by unpaired T-test.

To determine if the upregulation of Chrdl1 was astrocytic, and not due to upregulation from a non-astrocyte cell type, we used FISH in layers 2/3 of the visual cortex to analyze Chrdl1 mRNA overlap with markers for astrocytes (Slc1a3, also known as Glutamate Aspartate Transporter, GLAST) and neurons (Tubb3) in the peri-infarct area. This agreed with our previous study^20^ where we found that Chrdl1 was mainly expressed by astrocytes, and we verified that ischemic injury does not trigger upregulation of Chrdl1 from a non-astrocyte cell population. We found that Chrdl1 mRNA was overlapping with GLAST in the acute phase (Fig. 2a,d ipsi: sham, 74.39 ± 3.75%, PT 80.73 ± 0.76%), the sub-acute phase (Fig. 2b,e ipsi: sham 78.91 ± 1.33%, PT 88.63 ± 1.98) and the chronic phase (Fig. 2c,f ipsi: sham, 63.82 ± 0.97%, PT, 67.88 ± 1.06%) with no significant differences between animals subjected to PT or sham surgeries (Table S1). We found that at every time point, nearly 100% of GLAST + cells in WT mice express Chrdl1 in both sham and PT conditions (Fig. S2b–d). We also assessed Chrdl1 signal from individual astrocytes, as described before^28^, and found that individual astrocytes (GLAST + cells) significantly increased their Chrdl1 expression at 24 h (Fig. 2g, ipsi: sham 11.68 ± 4.28, PT 38.71 ± 8.91) and 7 days after PT (Fig. 2h, ipsi: sham 17.51 ± 5.41, PT 61.36 ± 7.72) while at 30 days post-PT the expression of Chrdl1 obtained from individual astrocytes returned to physiological levels (Fig. 2i, ipsi: sham 19.66 ± 3.39, PT 26.67 ± 6.32), in accordance with our results looking at Chrdl1 expression in cortical layers 2/3 of tissue (Fig. 1). These results suggest that the upregulation of astrocytic Chrdl1 after ischemic injury may trigger mechanisms that hinder synaptic plasticity, potentially obstructing synaptic regeneration and hampering recovery.

We also analyzed the expression of the astrocyte-enriched gene Slc1a3 (GLAST) to see whether the changes in expression that we observed in Chrdl1 were specific to Chrdl1 or shared by other astrocyte genes. GLAST was chosen due to its important roles in glutamate uptake in the context of excitotoxic injuries such as ischemia^29^. We found that GLAST was stable in the peri-infarct area (Fig. S1a,b ipsi: PT, 1.23 ± 0.06-fold compared to sham) and in the homologous contralateral region during the acute phase (Fig. S1a,b, contra: PT 1.18 ± 0.26-fold compared to sham). During the sub-acute phase, and similar to what we observed with the expression of Chrdl1, GLAST expression increased in the peri-infarct area (Fig. S1c,d, ipsi: PT, 1.63 ± 0.21-fold compared to sham), while in the homologous contralateral region there was no significant upregulation (Fig. S1c,d contra: PT, 1.55 ± 0.10-fold compared to sham). During the chronic phase, expression of GLAST returned to physiological levels (Fig. S1e,f, ipsi: PT 0.62 ± 0.18-fold compared to sham; contra: PT 0.73 ± 0.14-fold compared to sham). These results demonstrate that both Chrdl1 and GLAST expression are upregulated in response to ischemic injury but with distinct time courses, with Chrdl1 upregulation showing more rapid changes than GLAST in response to the same sort of injury.

### Reactive astrogliosis is not impaired in Chrdl1 KO mice after PT injury

**Figure 3:**
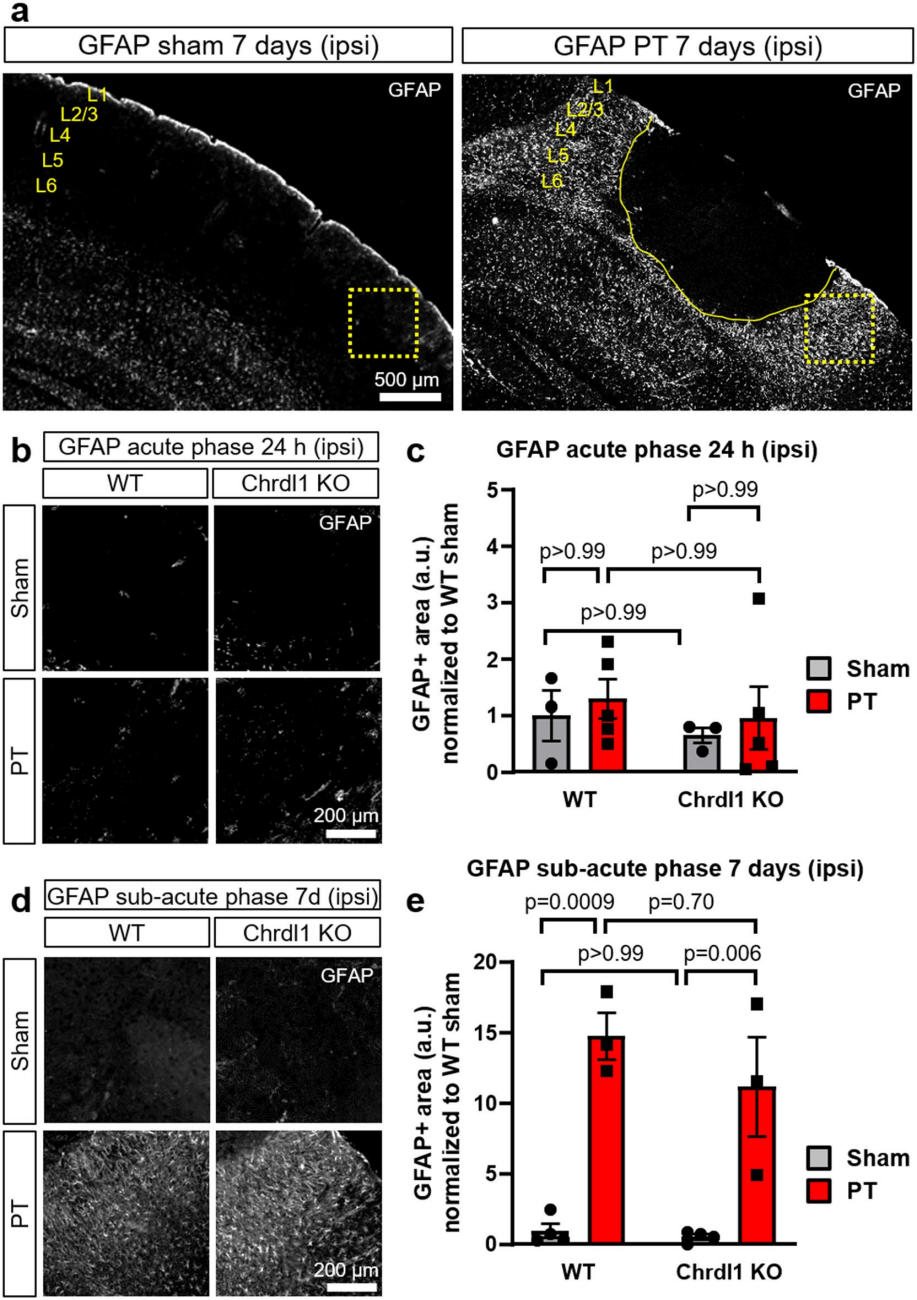
Reactive astrogliosis is not impaired in Chrdl1 KO mice after PT injury. (**a**) Representative images of the upper layers of the visual cortex of a WT mouse 7 days after sham surgery or PT for GFAP protein staining. The area in a dashed box is the region where analysis was performed. Core of the ischemic injury is delineated by the solid yellow line. Scale bar 500 µm. (**b**) Representative images of the peri-infarct area of WT and Chrdl1 KO mice 24 h after PT or sham surgery immunostained for GFAP. (**c**) Quantification of GFAP + stained area normalized to WT sham. WT sham N = 3, WT PT N = 5, Chrdl1 KO sham N = 3 and Chrdl1 KO PT N = 5 mice. (**d**,**e**) Same as (**a**,**b**) 7 days after PT or sham surgery immunostained for GFAP. WT sham N = 4, WT PT N = 3, Chrdl1 KO sham N = 4 and Chrdl1 KO PT N = 3 mice. Statistics by two-way ANOVA. Scale bar 200 µm.

As a consequence of ischemic injury, astrocytes become reactive in the peri-infarct area and undergo molecular, morphological and functional changes that drive the formation of a border surrounding the core of the injury^30^. Glial fibrillary acidic protein (GFAP) is a cytoskeletal protein that is upregulated in astrocytes in response to different types of CNS injury, and it is an indicator of reactive astrogliosis^30^. In a previous study, elimination of the plasticity-limiting neuronal molecular PirB led to reduction in GFAP levels, indicating a decline in astrocyte reactivity^22^. We assessed whether Chrdl1 played a role in attenuation or intensification of reactive astrogliosis in the peri-infarct area by performing PT in male Chrdl1 KO (-/y) and WT (+ /y) mice and analyzing GFAP stained area in the peri-infarct region at different post-injury stages. Overall we found a significant increase of GFAP restricted to the peri-infarct area at 7 days post-injury (Fig. 3a), when GFAP upregulation has been previously reported^31^. During the acute phase we did not detect significant increases in GFAP in either WT or Chrdl1 KO peri-infarct areas (Fig. 3b,c 24 h post-injury, WT: PT 1.31 ± 0.35-fold compared to WT sham; Chrdl1 KO: sham 0.66 ± 0.14-fold compared to WT sham; Chrdl1 KO: PT 0.96 ± 0.56-fold compared to WT sham). During the sub-acute phase, we detected an increased intensity in GFAP staining which was significant, but independent of Chrdl1 expression (Fig. 3d,e, 7 days post-injury, WT: PT 14.82 ± 1.65-fold compared to WT sham; Chrdl1 KO: sham 0.56 ± 0.19-fold compared to WT sham; Chrdl1 KO PT 11.22 ± 3.50-fold compared to WT sham). There were no variations in GFAP levels in the contralateral hemisphere during the acute (Fig. S3a,b) or sub-acute phases (Fig. S3c,d). Our results indicate that Chrdl1 does not regulate GFAP, a protein involved in reactive astrogliosis and in the formation of the astrocytic border in certain pathological contexts^32^.

### Absence of Chrdl1 does not affect the microglia response after PT injury

**Figure 4:**
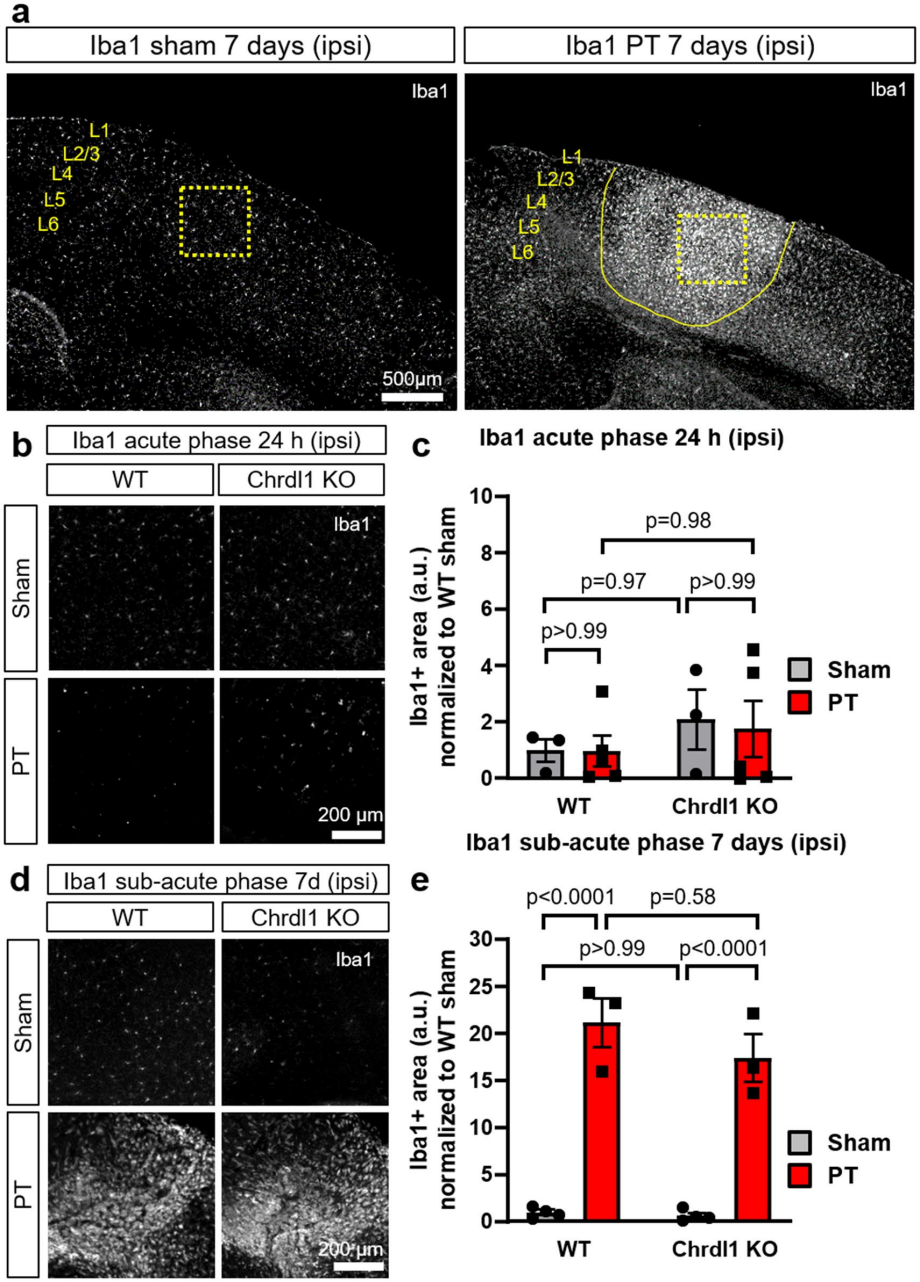
Absence of Chrdl1 does not affect the microglia response after PT injury. (**a**) Representative image of the upper layers of the visual cortex of a WT mouse 7 days after sham surgery or PT for Iba1 protein staining. The area in a dashed box is the region where analysis was performed. Core of the ischemic injury is delineated by the solid yellow line. Scale bar 500 µm. (**b**) Example images of the injury core of WT and Chrdl1 KO mice 24 h after PT or sham surgery immunostained for Iba1. (**c**) Quantification of Iba1 + stained area normalized to WT sham. WT sham N = 3, WT PT N = 5, Chrdl1 KO sham N = 3 and Chrdl1 KO PT N = 5 mice. (**d**,**e**) Same as (**a**,**b**) 7 days after PT or sham surgery immunostained for Iba1. WT sham N = 4, WT PT N = 3, Chrdl1 KO sham N = 4 and Chrdl1 KO PT N = 3 mice. Statistics by two-way ANOVA. Scale bar 200 µm.

Microglia have important roles in neuroinflammation; however, it is unclear what determines their beneficial or detrimental functions in response to ischemic injury. The use of Iba1 has been previously used in the context of stroke to study microglia activation, and it has been shown that in response to ischemia, Iba1 + microglial cells increase in the core of the injury which indicates activation of a microglial response^33^. Thus, we used Iba1 as an appropriate marker to identify the presence of microglia infiltrating to the core of the ischemic injury (7 days post-injury, sub-acute phase Fig. 4a). We assessed if there was a role for Chrdl1 in microglia activation by performing Iba1 staining of WT and Chrdl1 KO tissue after PT. In the core of the injury Iba1 signal was not evident during the first 24 h post-injury corresponding to the acute phase (Fig. 4b,c, 24 h post-injury, WT: PT 0.97 ± 0.55-fold compared to WT sham; Chrdl1 KO: sham 2.09 ± 1.06-fold compared to WT sham; Chrdl1 KO PT 1.76 ± 0.99-fold compared to WT sham), but it was apparent at 7 days post-injury, during the sub-acute phase, in both WT and Chrdl1 KO mice (Fig. 4d,e, 7 days post-injury, WT: PT 21.18 ± 2.02-fold compared to WT sham; Chrdl1 KO: sham 0.67 ± 0.29-fold compared to WT sham; Chrdl1 KO PT 17.42 ± 2.51-fold compared to WT sham). In both WT and Chrdl1 KO mice, differences in microglia recruitment were not observed in the contralateral hemisphere during the acute phase (Fig. S4a,b) or the sub-acute phase (Fig. S4c,d). Our results indicate that Chrdl1 does not have a large effect on the microglia response to ischemic injury.

### Absence of Chrdl1 does not affect PT injury volume

**Figure 5:**
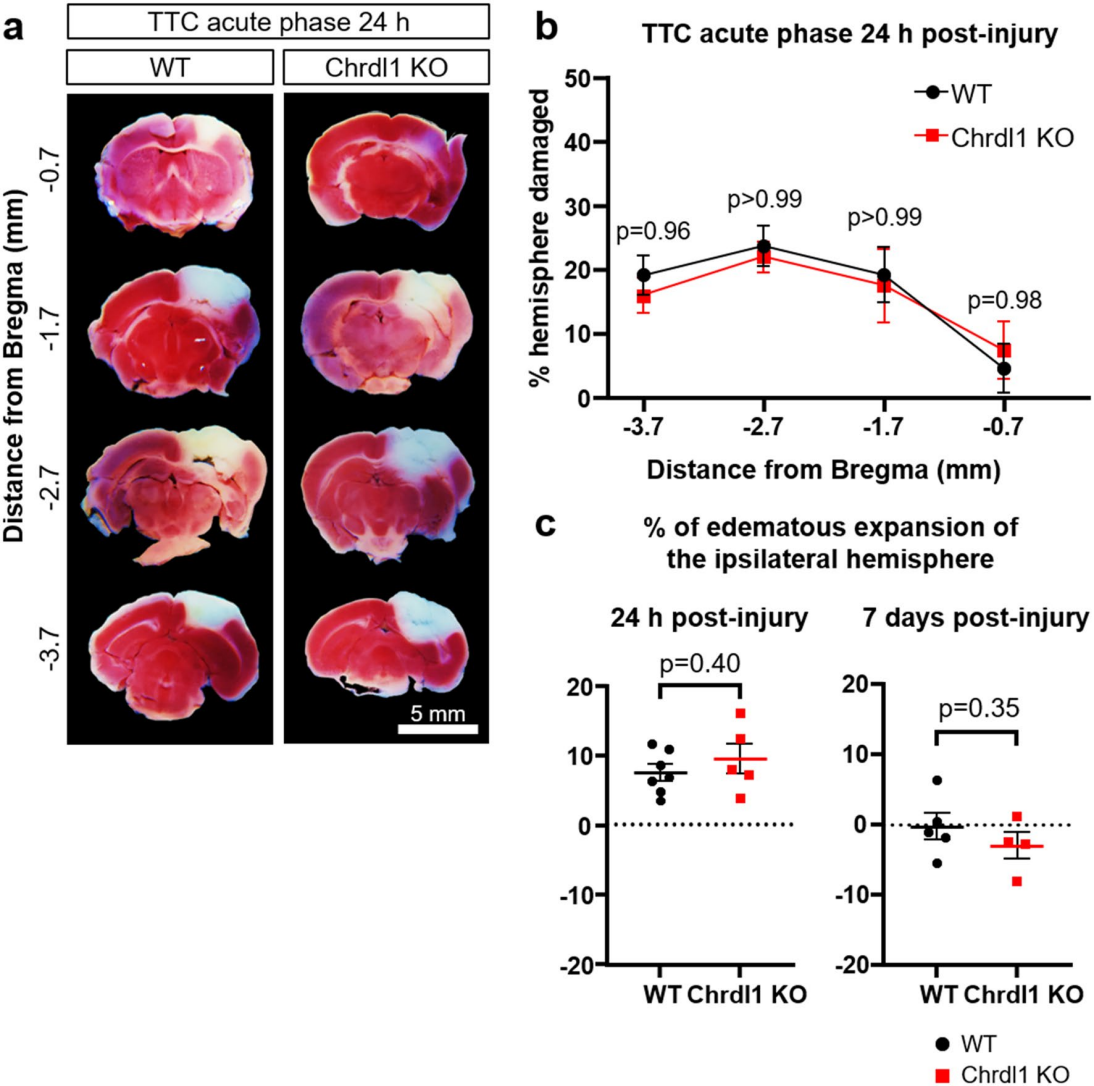
Absence of Chrdl1 does not affect PT injury volume. (**a**) Representative images of 1 mm-thick coronal sections of WT and Chrdl1 KO mouse brains 24 h after PT stained with TTC, corresponding to 0.7, 1.7, 2.7 and 3.7 mm posterior from Bregma. The dead tissue (brain regions infarcted) is not stained by TTC and appears white, whereas the rest of the tissue turns a shade of red. Scale bar 5 mm. (**b**) Quantification of the volume of the injury as percentage of the total volume of the ipsilateral hemisphere. WT N = 7, Chrdl1 KO N = 5 mice. Statistics by two-way repeated measures ANOVA. (**c**) Post-injury edematous expansion of the ipsilateral hemisphere represented as % relative to the total volume of the contralateral hemisphere at 24 h or 7 days post-injury. 24 h post-injury: WT N = 7, Chrdl1 KO N = 5 mice; 7 days post-injury: WT N = 5, Chrdl1 KO N = 4 mice. Statistics by unpaired T-test.

One factor that determines the severity of the injury is the size of the brain region affected by the deprivation of blood supply. Increasing plasticity can help repair and remap neuronal circuits that were damaged during the ischemic episode^34^. It has been shown that manipulating astrocytic or neuronal plasticity-regulating molecules contributes to reducing the size of the injury and improved functional recovery^19,22^. In our previous study we found that constitutive Chrdl1 KO mice display enhanced experience-dependent plasticity in the visual system^20^, thus we hypothesized that Chrdl1 KO mice would show decreased injury volume after an ischemic lesion. However, absence of Chrdl1 did not affect the volume of the injury during the acute phase (Fig. 5a,b, 24 h post-injury, WT: − 0.7 mm from Bregma 4.64 ± 3.74%, − 1.7 mm from Bregma 19.29 ± 4.29%, − 2.7 mm from Bregma 23.84 ± 3.19%, − 4.7 mm from Bregma 19.24 ± 2.99%, Chrdl1 KO: − 0.7 mm from Bregma 7.47 ± 4.58%, − 1.7 mm from Bregma 17.60 ± 5.75%, − 2.7 mm from Bregma 22.06 ± 2.45%, − 4.7 mm from Bregma 16.04 ± 2.77%). It is important to evaluate brain swelling derived from ischemic edema that can skew analysis of the injury volume^35^. We did not find differences in the edematous expansion between WT and Chrdl1 KO mice at 24 h or 7 days post-PT (Fig. 5c, 24 h post-injury, WT: 7.72 ± 1.16%, Chrdl1 KO: 9.69 ± 2.13%; 7 days post-injury, WT: − 0.24 ± 1.91%, Chrdl1 KO: − 2.99 ± 1.9%). Due to the limitations of the TTC staining protocol, the core of the injury in WT and Chrdl1 KO mice appears reduced at 7 days post-injury compared to 24 h post-injury. This is likely due to the infiltration of other living cells in the core of the injury (such as microglia) which enables the staining of the tissue by the TTC dye. Nevertheless, the injury volume during the sub-acute phase appeared unaltered in the absence of Chrdl1 (Fig. S5a,b, 7 days post-injury, WT: − 0.7 mm from Bregma 0.00 ± 0.00%, − 1.7 mm from Bregma 6.65 ± 3.34%, − 2.7 mm from Bregma 8.56 ± 3.26%, − 4.7 mm from Bregma 6.54 ± 1.61%, Chrdl1 KO: − 0.7 mm from Bregma 0.00 ± 0.0, − 1.7 mm from Bregma 7.96 ± 2.41%, − 2.7 mm from Bregma 7.33 ± 2.63%, − 4.7 mm from Bregma 4.86 ± 2.42%). As expected, mice subjected to sham surgeries did not show any injury (Fig. S5c,d). This demonstrates that Chrdl1 does not impact the overall size of the core of the injury. However, use of TTC staining does not inform about the potential survival of some cells in the core of the injury.

### Cell death is reduced in Chrdl1 KO mice after PT injury

**Figure 6:**
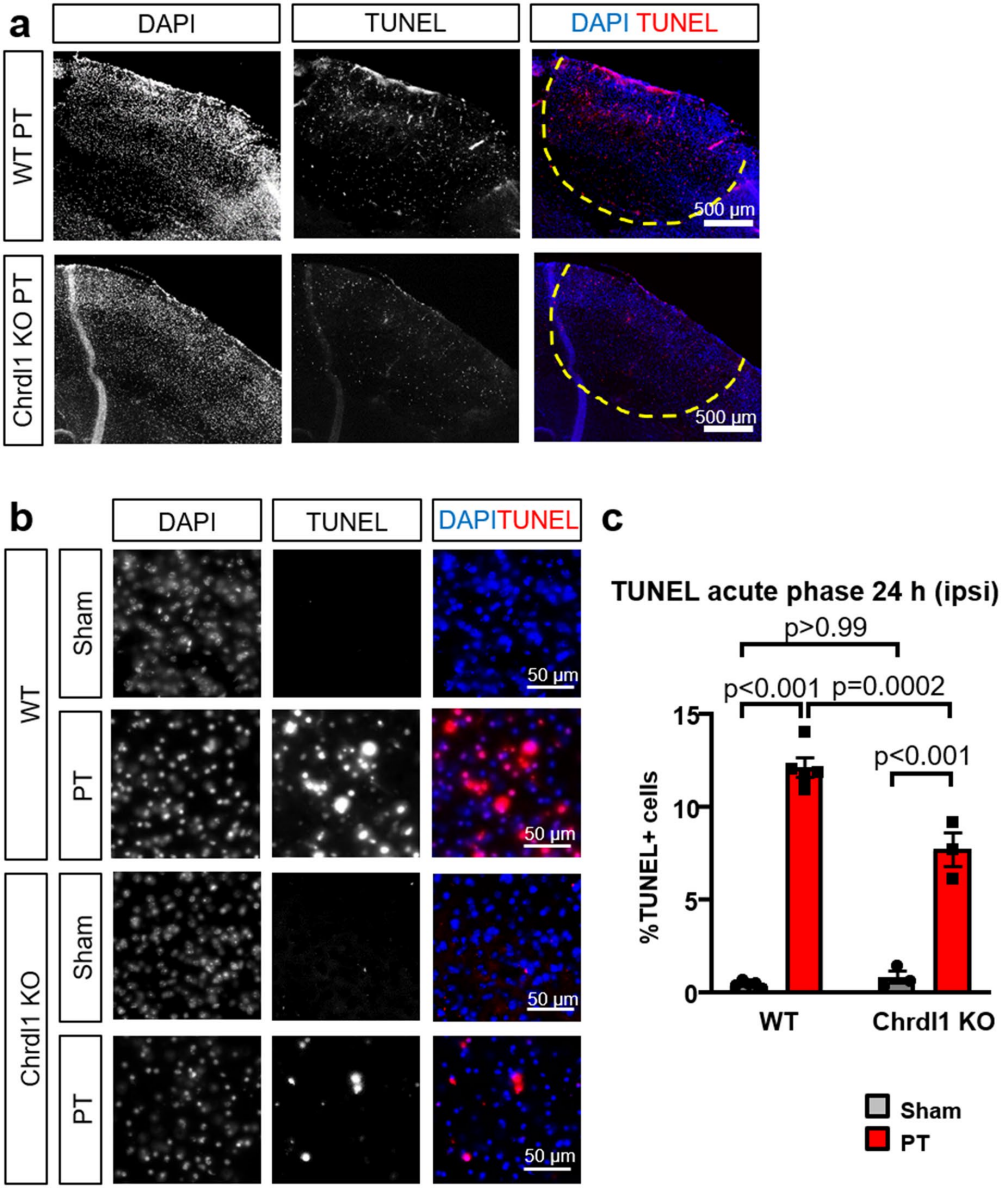
Cell death is reduced in Chrdl1 KO mice after PT injury. (**a**) Representative images of the upper layers of the visual cortex of a WT and a Chrdl1 KO mouse 24 h after PT showing TUNEL staining. Core of the ischemic injury is delineated by the solid yellow line. Analysis was performed in random fields of view within the core of the injury. Scale bar 500 µm. (**b**) Example images of TUNEL staining of WT and Chrdl1 KO core (ipsi) in layers 2/3 of the visual cortex. Scale bar 50 µm (**c**) Quantification of the % of cells stained with TUNEL. WT sham N = 5, WT PT N = 5, Chrdl1 KO sham N = 3, Chrdl1 KO PT N = 3 mice. Statistics by two-way ANOVA.

Although the core of the injury is generally considered tissue that cannot be rescued, some cells may survive and be the basis for future recovery. Without intervention damage from the core can extend into the peri-infarct area, aggravating the functional outcomes. Thus, we investigated the extent of cell death in the core of the injury of Chrdl1 KO compared to WT mice during the acute phase, the post-injury stage when acute cell death occurs^7^. To do this, we assessed in situ cell death using TUNEL technology, and found that Chrdl1 KO mice displayed reduced apoptotic cell death levels compared to WT 24 h post-injury as quantified by the % of TUNEL positive cells within the core of the injury (Fig. 6, WT sham 0.46 ± 0.08%; WT PT 12.15 ± 0.53%; Chrdl1 KO sham 0.83 ± 0.33%; Chrdl1 KO PT 7.72 ± 0.89%). No apoptotic cell death was observed in the contralateral hemisphere (Fig. S6). Although our results indicate a small difference, they suggest that absence of Chrdl1 may attenuate injurious signaling from the core into the peri-infarct area, where there is tissue that holds potential for recovery and repair.

### Absence of Chrdl1 prevents spine loss in the peri-infarct area

**Figure 7:**
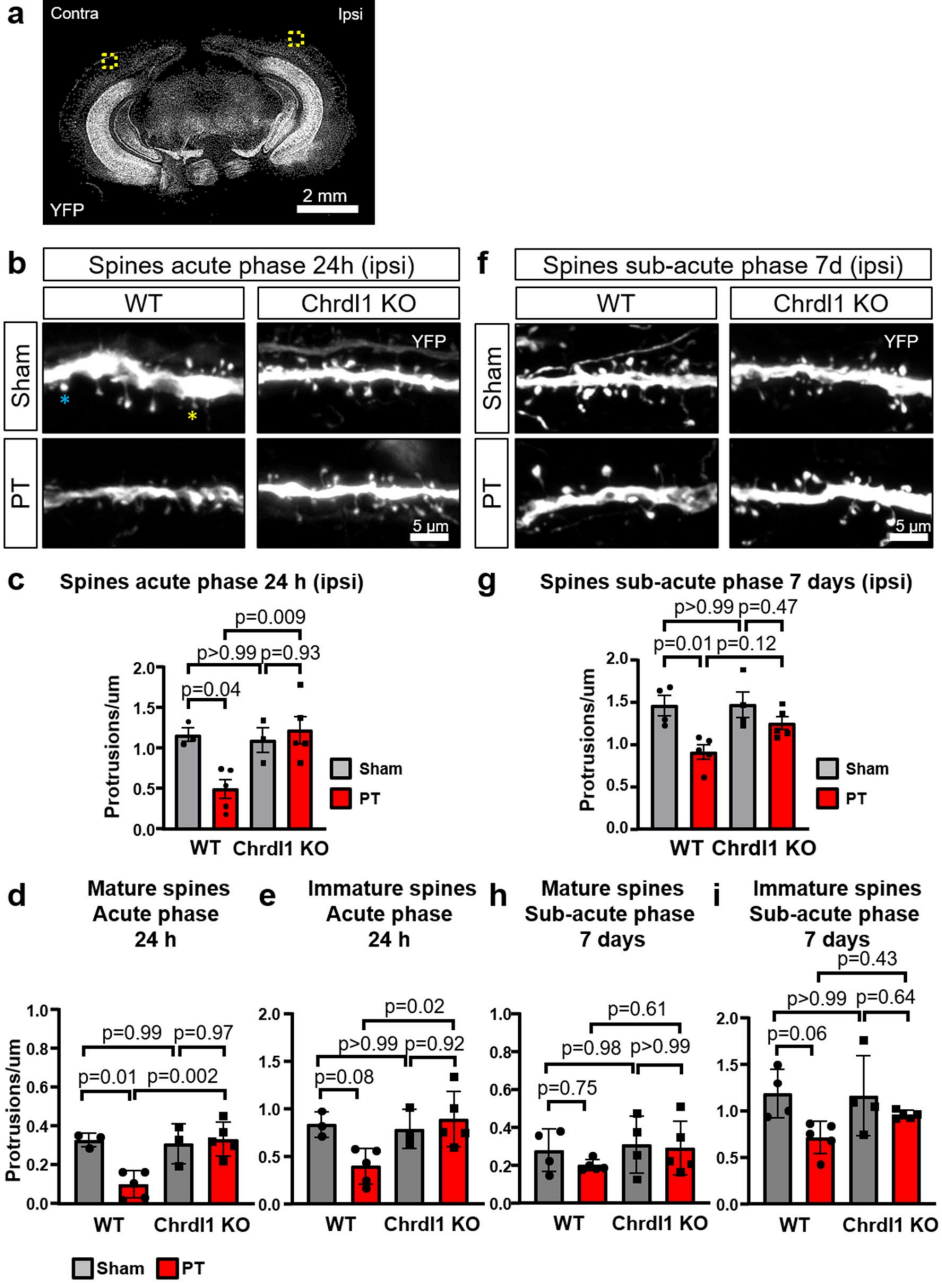
Absence of Chrdl1 prevents spine loss in the peri-infarct area. (**a**) Overview of a coronal brain slice from a YFP-expressing male mouse. Dashed boxes indicate the ipsilateral (ipsi) and contralateral (contra) regions where dendritic spines were imaged and analyzed. Scale bar 2 mm. (**b**) Representative images of layer 5 neuron secondary dendrites expressing YFP in WT or Chrdl1 KO mice, in layers 2/3 of the visual cortex 24 h after PT or sham surgeries. The blue star indicates an example of a spine of mature morphology, and the yellow star indicates an example of a spine of immature morphology. Scale bar 5 µm. (**c**) Quantification of number of spines per µm on dendrites in layers 2/3 in the peri-infarct area of the visual cortex of mice 24 h after PT or sham surgery. WT sham N = 3, WT PT N = 5, Chrdl1 KO sham N = 3, Chrdl1 KO PT N = 5 mice. Statistics by one-way ANOVA. (**d**) Quantification of spines with mature morphology in the peri-infarct area of dendrites in layers 2/3 of the visual cortex of mice 24 h after PT or sham surgery. WT sham N = 3, WT PT N = 5, Chrdl1 KO sham N = 3, Chrdl1 KO PT N = 5 mice. Statistics by one-way ANOVA. (**e**) Quantification of spines with immature morphology in the peri-infarct area of dendrites in layers 2/3 of the visual cortex of mice 24 h after PT or sham surgery. WT sham N = 3, WT PT N = 5, Chrdl1 KO sham N = 3, Chrdl1 KO PT N = 5 mice. Statistics by one-way ANOVA. (**f**) Same as (**b**), 7 days after PT or sham surgery. Scale bar 5 µm. (**g**) Same as (**c**), 7 days after PT or sham surgery. WT sham N = 4, WT PT N = 5, Chrdl1 KO sham N = 4, Chrdl1 KO PT N = 5 mice. Statistics by one-way ANOVA. (**h**) Same as (**d**), 7 days after PT or sham surgery. WT sham N = 4, WT PT N = 5, Chrdl1 KO sham N = 4, Chrdl1 KO PT N = 5 mice. Statistics by one-way ANOVA. (**i**) Same as (**e**), 7 days after PT or sham surgery. WT sham N = 4, WT PT N = 5, Chrdl1 KO sham N = 4, Chrdl1 KO PT N = 5 mice. Statistics by one-way ANOVA.

Immediately after ischemia, there is an acute loss of dendritic spines in the peri-infarct area in WT mice. The decreased spine density is severe during the acute phase, with signs of potential recovery during the sub-acute phase^25^. The absence of Chrdl1 does not affect spine density or morphology in uninjured adult mice^20^, but we hypothesized that it may have an effect in response to ischemic injury, as was observed in other studies where plasticity-regulating proteins were eliminated^36^. To this end, WT and Chrdl1 KO mice where layer 5 neurons express the fluorescent protein YFP were subjected to sham surgeries or PT in the visual cortex, and spine density of secondary dendrites extending into layers 2/3 of the visual cortex were analyzed (Fig. 7a). WT mice subjected to PT injury displayed severe loss of spines in the peri-infarct area during the acute phase (Fig. 7b,c, 24 h post-injury: WT sham 1.17 ± 0.08 protrusion/µm; WT PT, 0.50 ± 0.11 protrusion/µm), a well-documented consequence of ischemic lesions^25,37,38^. Interestingly, Chrdl1 KO mice did not show the characteristic ischemic-induced decrease in spine density during the acute phase (Fig. 7b,c, 24 h post-injury: Chrdl1 KO sham 1.10 ± 0.15 protrusion/µm; Chrdl1 KO PT 1.22 ± 0.17 protrusion/µm), suggesting that absence of Chrdl1 prevents the initial degeneration of spines in the peri-infarct area. We observed that spine loss in the peri-infarct area was persistent in WT mice during the sub-acute phase, though not as severe as initially observed (Fig. 7f,g, 7 days post-injury: WT sham 1.46 ± 0.12 protrusion/µm; WT PT 0.91 ± 0.08 protrusion/µm). During the sub-acute phase, Chrdl1 KO mice displayed spine numbers similar to those mice that went under sham surgeries (Fig. 7f,g, 7 days post-injury: Chrdl1 KO sham 1.47 ± 0.15 protrusion/µm; Chrdl1 KO PT 1.25 ± 0.08 protrusion/µm), suggesting that absence of Chrdl1 protects from spine loss rather than delaying the neurodegenerative processes that drive spine elimination after injury. Neither WT nor Chrdl1 KO mice showed variation in spine density in the contralateral hemisphere (Fig. S7a,b,e,f).

Previous studies have extensively investigated the link between morphology and maturity of dendritic spines^39,40^. It is established that dendritic spines with mushroom morphologies are characteristic of mature spines and considered to establish strong synaptic connections, as opposed to those with thin and stubby morphologies that are highly dynamic and predominant during developmental stages and plasticity processes^39,40^. One possibility is that Chrdl1 KO mice experience spine loss with a fast turnover during the early acute phase (first 24 h post-injury), in which case an increase in immature spine morphologies would be expected compared to mice that went under sham surgeries. We found that WT mice showed a significant decrease in mature spines after injury (Fig. 7d, WT sham 0.33 ± 0.02 protrusion/µm; WT PT 0.10 ± 0.03 protrusion/µm). Chrdl1 KO did not show any alterations in mature spine density in the peri-infarct area during the acute phase (Fig. 7d, Chrdl1 KO sham 0.31 ± 0.06 protrusion/µm; Chrdl1 KO PT 0.33 ± 0.04 protrusion/µm). During the acute phase, WT mice also showed a decrease in immature spine density (Fig. 7e, WT sham 0.84 ± 0.08 protrusion/µm; WT PT 0.40 ± 0.08 protrusion/µm), whereas Chrdl1 KO mice had a constant density of immature spines after injury compared to sham animals (Fig. 7e, Chrdl1 KO sham 0.79 ± 0.12 protrusion/µm; Chrdl1 KO PT 0.89 ± 0.13 protrusion/µm). During the sub-acute phase, the WT mice showed restoration of mature spine density (Fig. 7h, WT sham 0.28 ± 0.06 protrusion/µm; WT PT 0.20 ± 0.01 protrusion/µm), while the Chrdl1 KO mice showed no changes in the mature spine density (Fig. 7h, Chrdl1 KO sham 0.31 ± 0.08 protrusion/µm; Chrdl1 KO PT 0.29 ± 0.06 protrusion/µm). On the other hand, density of immature spines in the WT mice during the sub-acute phase remained low (Fig. 7i, WT sham 1.18 ± 0.13 protrusion/µm; WT PT 0.71 ± 0.08 protrusion/µm). During the sub-acute phase Chrdl1 KO mice did not experience reduction of immature spines as the density of the spines with immature morphology remained at comparable levels to the acute phase (Fig. 7i, Chrdl1 KO sham 1.16 ± 0.21 protrusion/µm; Chrdl1 KO PT 0.96 ± 0.02 protrusion/µm). There were no significant morphological changes in the contralateral hemisphere of WT or Chrdl1 KO in response to ischemic insult (Fig. S7c,d,g,h). This indicates that the absence of Chrdl1 protected the peri-infarct neurons from the typical spine loss observed in WT mice in response to ischemic injury, maintaining consistent spine density and steady proportion of mature and immature spine morphologies.

## Discussion

In this study we investigated the contribution of the astrocyte-enriched synapse-regulating protein Chrdl1 to the response to PT injury. We found that Chrdl1 is upregulated after injury, and that removal of Chrdl1 prevents neuronal dendritic spine loss that is characteristic of the acute phase after injury and reduces cell death during the early stages post-injury. We further found that this protective role of Chrdl1 is independent of reactive gliosis and does not impact on the initial size of the lesion.

We found that Chrdl1 expression is upregulated in the acute phase after PT in the peri-infarct area and in the contralateral hemisphere, and that the upregulation is persistent during the sub-acute phase only in the peri-infarct area, with return to physiological levels at later time points during the chronic phase (Figs. 1b–g, 2g–i). Contralateral alterations are possible in response to a focal injury as observed before^41^ but the mechanisms are yet to be fully described. The expression of Chrdl1 is astrocyte-enriched and ischemic conditions do not trigger expression in non-astrocyte cell populations (Fig. 2). We also found that GLAST (Slc1a3) is increased in expression in the sub-acute phase, but not during the acute or chronic phases (Fig. S1). In ischemia there is a massive release of glutamate into the synaptic cleft that ultimately triggers excitotoxicity and cellular death, and astrocytes play important roles in re-uptake of excessive glutamate including through GLAST^42^. The differential temporal changes of Chrdl1 and GLAST expression in response to PT suggest that other astrocyte factors may also display differential temporal regulation in response to the same kind of injury. In the future it will be important to assess astrocyte gene expression during the acute, sub-acute and chronic phases, as they may dictate the endogenous plasticity levels that determine functional recovery and circuit remodeling in response to injury.

The increased expression of Chrdl1 may represent an inhibitory role of astrocytes in response to ischemic conditions, as Chrdl1 appears to limit plasticity during the sub-acute phase (Fig. 7). This suggests that eliminating Chrdl1 further enhances post-injury endogenous plasticity during the sub-acute phase. Studies in other injury and disease settings found that Chrdl1 is upregulated in an animal model of spinal cord injury^43^, while in chronic disease animal models, such as Huntington’s disease and amyotrophic lateral sclerosis, there is no alteration in Chrdl1 expression^44,45^. This suggests that the upregulation of Chrdl1 is context-specific to CNS acute injury, and that the functional role of Chrdl1 that we show here is limited to these settings. In future it will be important to assess Chrdl1 expression in human tissue after acute injury such as ischemic stroke. The results presented here relate to RNA levels of Chrdl1 and suggest that protein secretion may be increased in response to ischemic injury. However, due to technical limitations, protein levels of secreted Chrdl1 in the peri-infarct area have not been possible to assess in the present work, and it will be a necessary analysis for future studies.

Glial cells respond to diverse types of injury and disease through a series of molecular and morphological changes that group under the term reactive gliosis. In the case of astrocytes, reactive astrogliosis in its most severe form leads to the formation of the glial scar^30^. A previous study showed that elimination of plasticity-limiting molecules, such as PirB, leads to reduction in GFAP levels, thus decreasing astrocyte activation^22^. According to our results based on GFAP immunohistochemistry, Chrdl1 absence does not influence reactive astrogliosis (Fig. 3, Fig. S3), suggesting that formation of an astrocytic border surrounding the core of the injury is Chrdl1-independent in the context of this model. This can explain why the absence of Chrdl1 does not affect the volume of the injury in Chrdl1 KO mice when compared to WT mice (Fig. 5, Fig. S5), as reactive astrogliosis and the associated border formation is not affected by knocking out Chrdl1. The effects of Chrdl1 appear to be related to neuronal plasticity mechanisms in response to external stimuli, such as sensory deprivation^20^ or injury, rather than intrinsic mechanisms of astrocytes themselves that lead to reactive astrogliosis and formation of the astrocytic border. Further experiments to analyze other reactive markers like Lcn2 or Serpina3n among others^26^ will clarify whether Chrdl1 regulates astrogliosis in ischemic conditions.

The immune response in ischemic injury is regulated by various glial cell types that also interact among themselves to regulate inflammation, and these glia-mediated mechanisms can exert both beneficial and detrimental effects^16^. Astrocytes and microglia are two important components of the neurovascular unit, and both are activated in response to ischemia, as well as other types of CNS injuries^46^. It has been previously shown that activated microglia can induce reactive astrogliosis with neurotoxic effects^47^, but the mechanisms that regulate microglia activation are still not completely clear. In the present study we found that absence of Chrdl1 does not affect microglia activation, as demonstrated by Iba1 immunostaining (Fig. 4, Fig. S4). This suggests that Chrdl1 does not play a role in the inflammatory response in this injury model, an important feature that leads to further damage beyond the core and the peri-infarct areas, contributing to the extension of the injury and worse outcomes. Therefore, the beneficial effects of Chrdl1 removal on dendritic spine number does not appear to be related to inflammation or astrogliosis. A previous study demonstrated that overexpression of Noggin, a BMP (Bone Morphogenetic Protein) antagonist similar to Chrdl1, promoted increased Iba1 + microglia during the sub-acute phase in a mouse model of pMCAO (permanent MCAO)^48^, which promoted reduction in the infarct volume. In future it will be interesting to study the effects of Chrdl1 overexpression on the microglial response and whether this may influence the injury volume.

During the first hours and days following injury, cells in the core and in the peri-infarct area follow apoptotic cascades triggered by excitotoxic events and energy failure due to the interruption of blood flow^49^. Cell survival and the volume of the injury have important implications in the functional outcome from ischemic conditions. Survival of neuronal and non-neuronal cells in the core of the injury can be crucial for repair and recovery, as that prevents release of pro-apoptotic proteins that contribute to increased injury volume^50^. Normally, larger injury sizes are more likely to have fatal outcomes^51^. Lack of Chrdl1 promotes enhanced experience-dependent plasticity in the brains of mice^20^ and so we hypothesized that absence of Chrdl1 may facilitate recovery from injury through reduction of the core volume, as has been shown for other plasticity limiting molecules^22^, and thus, decline in cell death. We found, however, that absence of Chrdl1 did not have any effects on the volume of the injury (Fig. 5, Fig. S5), but Chrdl1 KO mice showed a significant reduction, albeit small, in cell death during the acute phase (Fig. 6). This indicates that absence of Chrdl1 may have beneficial roles during the early post-injury stages. This is in contrast to the study where Noggin was overexpressed, promoting reduction in injury size during the sub-acute phase and improving behavioral outcomes^48^, supporting the hypothesis that different BMP inhibitors may display differences in their mode of action. Interestingly, a previous study on thrombospondin-1 and -2, two astrocyte-secreted synaptogenic proteins, showed that their absence did not play a role in the size of the injury, but impaired functional recovery^19^. However some studies suggest that injury size does not correlate with functional deficits^52^, in contrast with reports from other groups^51^. Our results indicate that although Chrdl1 does not influence injury volume, it may limit apoptotic signaling pathways at early stages post-injury. In the future it will be interesting to assess the levels of other proteins that have been previously identified to regulate the size of the injury and cell death during ischemic conditions, and assess whether their expression is altered in Chrdl1 KO mice to trigger mechanisms that maintain injury volume while promoting cell survival.

In response to ischemic conditions neurons in the peri-infarct region undergo deep remodeling, featuring spine loss and morphological modifications which are especially severe during the acute phase^25^. During later stages post-injury, spontaneous mechanisms promote spinogenesis^6^, but those mechanisms remain unclear. Since we found an increased expression of Chrdl1 in response to ischemia that may hinder synaptic remodeling, we hypothesized that eliminating Chrdl1 could protect spine loss or promote faster spine turnover to compensate for the ischemic damage. Chrdl1 KO mice did not show the characteristic spine loss in the peri-infarct area in response to ischemic lesion as we found no difference in dendritic spine density in the Chrdl1 KO mice between 24 h and 7 days post-PT (Fig. 7, Fig. S7). We did not observe any alteration in spine morphology, suggesting that absence of Chrdl1 protects spines from injury-driven pruning. Time- and region-dependent Chrdl1 upregulation may hinder remodeling of damaged circuits by preventing formation and/or restructuring of neuronal synapses during the sub-acute phase. In the future live imaging experiments will help to determine whether spines in Chrdl1 KO mice are stable and protected in response to ischemic lesion, or experience a rapid turnover after injury. Alternatively, elimination of Chrdl1 may be facilitating a delay in dendritic spine loss, which may offer a wider time window for recovery. Performing electrophysiological recordings from peri-infarct neurons in Chrdl1 KO will inform whether the remaining dendritic spines retain synaptic activity. Additionally, it will be important to perform behavioral analysis to assess if functional recovery is improved by blocking astrocyte-secreted Chrdl1 at different time points, or if the protection of spines observed in Chrdl1 KO mice offers a longer time window for recovery.

Our work shows that increased expression of Chrdl1 can be a detrimental contribution from astrocytes that impedes recovery by limiting plasticity and circuit remodeling. Elimination of Chrdl1 may promote protective effects in the visual cortex due to Chrdl1 role as a regulator of AMPAR-mediated synaptic maturation and synaptic plasticity as we previously reported^20^. It is known that different neuronal populations display differential surface levels of GluA2-containing AMPARs in ischemic-like conditions^53^, and this is linked to the differential vulnerability that neuronal subpopulations show to ischemic injury^53,54^. Thus, in the future it will be important to analyze the expression of Chrdl1 in other brain regions in response to ischemic conditions, as Chrdl1 may have variable effects on different neuronal populations. One possibility is that upregulation of Chrdl1 in response to injury is exclusive to upper layers of cortex and striatum where Chrdl1 is expressed in the healthy brain, and additional astrocyte-secreted factors may regulate post-injury plasticity in other brain regions.

An alternative explanation for the potential protective effect in absence of Chrdl1 after injury is through its role as an inhibitor of BMP signaling. It has been previously shown that administration of BMP7 24 h after ischemic injury improves behavioral recovery^55^. Post-injury upregulation of Chrdl1, a known BMP antagonist with affinity for BMP4, 6 and 7^56^, could be blocking the beneficial effects of BMPs after ischemic injury. Blockade of Chrdl1 after ischemia may therefore represent a new approach to promote the neurorestorative roles of BMPs. However, different BMPs may play diverse roles in injury, promoting recovery or delayed cell death. In a different study it was found that overexpressing the BMP antagonist Noggin had neuroprotective effects by reducing the size of the injury^48^. Noggin has high affinity for BMP4, like Chrdl1^56^. Therefore, it is important to determine the specific post-injury phase in which to manipulate Chrld1 in order to promote beneficial effects.

In conclusion, our results indicate that astrocyte-secreted factors play a role in structural plasticity and cell death after ischemic insult. The restricted regional expression of Chrdl1 suggests that alternative astrocyte-secreted factors that regulate plasticity and spine density in other brain regions may also mediate plasticity mechanisms and cell death in response to region-specific injury in a time-dependent manner, in an antagonistic or synergistic manner with Chrdl1. This study highlights the importance of dissecting astrocyte-mediated plasticity mechanisms to understand the limitations in circuit remodeling in response to injury and other pathologies of the CNS, and design new strategies to promote neuroprotection and recovery at different timepoints post-injury.

## Methods

All animal work was approved by the Institutional Animal Care and Use Committee (IACUC) of the Salk Institute for Biological Studies and performed in accordance with their guidelines. ARRIVE guidelines were followed in all animal experiments.

### Animals

Mice were housed in the Salk Institute animal facility at a light cycle of 12 h light:12 h dark, and access to water and food ad libitum.

Wild-type (WT) mice (C57BL/6J, Jax stock number 000664, RRID:IMSR_JAX:000664) were used for analysis of expression of Chrdl1 by in situ hybridization as described below, and to breed to Chrdl1 KO mice and Thy1-YFP-J mice (B6.Cg-Tg(Thy1-YFP)HJrs/J, Jax stock number 003782, RRID:IMSR_JAX:003782). For detailed procedure to generate Chrdl1 KO mice see reference^20^. For the generation of experimental mice heterozygous (+/−) Chrdl1 females (as Chrdl1 is in the X chromosome) were bred to WT (+/y) C57BL/6J males. All experiments were performed using male Chrdl1 KO (−/y) and WT (+/y) littermates. Thy1-YFP-J male mice were crossed with heterozygous Chrdl1 KO (+/−) females for the generation of Thy1-YFP-J Chrdl1 KO (-/y) and WT (+/y) males used for experiments (referred to as YFP-Chrdl1 KO and WT).

### Photothrombotic ischemic injury

Male mice at 4 months of age were placed on a stereotaxic frame and anesthetized by 2% isoflurane in oxygen by constant flow via nose cone. Mice were retro-orbitally injected with 10 mg/ml Rose Bengal (Fisher R323-25) in saline (0.9% NaCl) at a dose of 25 mg/kg. Rose Bengal was prepared fresh before surgeries and protected from light. Control mice subjected to sham surgeries were injected with the equivalent volume of saline (0.9% NaCl) with no Rose Bengal, and followed the same procedure. After injection, mice were prepared for surgery during the 5 min allowed for Rose Bengal dye to diffuse. Fur was removed and the scalp was disinfected with alternative swipes of 70% ethanol and betadine. During surgery body temperature was monitored and maintained at 37 ± 0.5 °C with a rectal probe and feedback-controlled heating pad. An incision through the midline with surgical scissors was made to expose the skull and locate Bregma. In this study, the brain region to apply photothrombosis is located at 3.28 mm posterior and 2.80 mm lateral to Bregma. After 5 min from Rose Bengal injection and location of stereotaxic coordinates, the brain region was illuminated through the skull for 10 min with a 520 nm diode laser (Thor labs) set at 2 mm diameter and 10 mW power. Skull was then washed with saline 0.9% NaCl and the incision was closed with Vetbond tissue adhesive (3 M 1469Sb). Triple antibiotic and 2% lidocaine were applied to the closed incision, and the mouse was returned to a clean cage. All analysis were made in the peri-infarct area which has been previously defined as the area contained within 200 µm from the border of the core of the injury^57,58^.

### Tissue collection and preparation

Brains were collected at 24 h, 7 days, or 30 days post-photothrombosis (PT) to study the acute, sub-acute or chronic phases, respectively. Mice were injected intraperitoneally with 100 mg/kg Ketamine (Victor Medical Company) and 20 mg/kg Xylazine (Anased) mix and subjected to transcardial perfusion. Perfusion was performed with PBS to obtain fresh frozen tissue for fluorescent in situ hybridization (FISH). Those brains were collected, embedded in OCT (Scigen 4583) and stored at − 80 °C until analysis. For the rest of experiments, perfusion was performed with PBS followed by 4% PFA (paraformaldehyde) to obtain fixed tissue. Brains were collected and stored in 4% PFA at 4 °C overnight, after which they were washed three times with PBS and transferred to 30% sucrose and stored for 3 days at 4 °C for cryoprotection. Those brains were embedded in TFM (General data healthcare TFM-5), frozen in dry ice/100% ethanol and stored at − 80 °C until analysis. For TTC staining, mice were euthanized by intraperitoneal injection of ketamine and xylazine mix and brains were collected without prior perfusion. For every experiment, a minimum of three brain sections were imaged per mouse, and a minimum of 3 mice per condition (sham or PT). N refers to the specific number of mice used in each experiment.

### Fluorescent in situ hybridization (FISH) and analysis

Coronal sections were obtained from WT C57BL/6 J mice that went under sham surgeries or PT and euthanized 24 h, 7 days or 30 days after surgery to analyze expression of Chrdl1. Sections were made at a thickness of 16 µm, cut with a cryostat (Hacker Industries OTF5000) at coordinates corresponding with the core of the injury (3.28 mm posterior and 2.80 mm lateral to Bregma). Fluorescent in situ hybridizations (FISH, ACDbio 320850) were performed according to manufacturer’s instructions with some modifications. Brain slices were pre-treated with Protease IV for 20 min. Probes used were Chrdl1 (ACDbio 442811), Slc1a3/GLAST (ACDbio 430781-C2), and Tubb3 (ACDbio 423391-C3). For negative control we used a 3-plex negative probe (ACDbio 320871) to determine the background fluorescence and the cut-off threshold to determine a positive stained cell (Fig. S2). For mounting, slices were applied with SlowFade gold antifade mountant with DAPI (Thermo Fisher Scientific S36939). On top of the sections, we used coverslips 22 mm × 50 mm 1.5 thickness and sealed with nail polish. Slc1a3 was imaged in channel 488, Chrdl1 in channel 550, and Tubb3 in channel 647. Peri-infarct region in the ipsilateral hemispheres, and the equivalent region in the contralateral hemispheres were imaged on a Zeiss LSM 700 confocal microscope using 20x/0.8 NA objective as 16-bit images at 1024 × 1024 pixels (pixel size 0.31 × 0.31 µm) as z-stacks of 5 slices with 10% overlap and a total thickness of 6.783 µm (time points 24 h and 7d post-PT) or using a confocal SP8 (Leica) using 20x/0.75NA objective as 16-bit images at 1024 × 1024 pixels (pixel size 0.31 × 0.31 µm) as z-stacks of 5 slices with 10% overlap and a total thickness of 6.783 µm (time point 30 days post-PT). Representative images are orthogonal projections. The cortical overview of Chrdl1 expression in Fig. 1h was taken on a Zeiss LSM 700 confocal microscope using a 20x/0.8 NA objective as a 16-bit image mosaic of stitched tiles with 10% overlap, image size 10,244 × 6556 pixels (pixel size 0.31 × 0.31 µm). Maximal intensity orthogonal projection images were produced with ImageJ for all subsequent analyses. The experimenter performing the analysis was blind to condition (sham or PT). For Fig. 1, fluorescence intensity was measured using ImageJ (NIH, RRID:SCR_003070), and calculated as mean intensity of the ROI multiplied by % of area stained. For Fig. 2d–f, cells co-expressing Chrdl1 signal and Tubb3 or Slc1a3 signal were counted using ImageJ multi-point function. In all cases, a consistent threshold appropriate for visualization of the stained area was applied, using a negative control to determine the cut-off for a positive stained cell. All results were normalized to the sham condition. In Fig. S2b, cells expressing Chrdl1 were counted and plotted as a % of Slc1a3 + cells. For Fig. 2g–i, we used a semi-automatic custom-made macro for ImageJ^28^. Astrocytes were identified using the Slc1a3 signal to create an ROI around each astrocyte. Then a consistent threshold was applied to the Chrdl1 probe signal across samples, and the threshold area was recorded for each astrocyte.

### GFAP and Iba1 staining

YFP-Chrdl1 KO and YFP-WT male mice were subjected to sham surgeries or PT, and perfused 24 h or 7 days later with PBS and 4% PFA to collect fixed tissue. Fixed brains were sliced in the cryostat at a thickness of 16 µm and stained for GFAP or Iba1. Upon collection, slices were incubated in blocking solution (5% goat serum, 0.3% Triton X-100 in PBS) for 1 h at room temperature. Slices were then incubated with rabbit polyclonal primary antibody anti-GFAP (Abcam ab7260, RRID:AB_305808) at a dilution 1:500 or rabbit polyclonal anti-Iba1 at a dilution 1:250 (Wako 016-20001, RRID:AB_839506) in antibody buffer (5% goat serum, 100 mM lysine, 0.3% Triton X-100 in PBS) overnight at 4 °C in a humidified chamber. For the negative controls, primary antibody was omitted (Fig. S3E). Slices were washed three times with PBS and incubated with secondary anti-rabbit Alexa 594 (Thermo Fisher Scientific A11012, RRID:AB_141359) in antibody buffer at a dilution of 1:500 for 2 h at room temperature. Slices were washed three times with PBS and mounted using SlowFade gold antifade mountant with DAPI (Thermo Fisher Scientific S36939), covered with coverslips 22 × 50 mm 1.5 thickness and sealed with nail polish. The core and the peri-infarct areas in the ipsilateral hemisphere (Figs. 3a and 4a) and equivalent regions in the contralateral hemisphere were imaged using a fluorescent microscope Zeiss Axio Imager.Z2. Images were taken using a 10x/0.45 NA objective as 16-bit mosaics of 9 tiles with 10% overlap. To measure GFAP signal, a region of interest (ROI) of 1000 × 1000 pixels (pixel size 0.645 × 0.645 µm) was selected in the peri-infarct area, and in the homologous contralateral area. For Iba1 signal measurements, an ROI in the core of the injury was selected. GFAP or Iba1 signal was measured using ImageJ and calculated as stain positive (GFAP + or Iba1 +) area of the ROI. All results were normalized to WT sham.

### 2,3,5-Triphenyltetrazolium chloride (TTC) staining

Chrdl1 KO and WT male mice were subjected to sham surgeries or PT and brains were extracted without prior perfusion 24 h or 7 days later after euthanasia by intraperitoneal injection of overdose of ketamine/xylazine mixture. Brains were cut in 1 mm thick slices and incubated in a pre-warm solution of 2% 2,3,5-Triphenyltetrazolium chloride (TTC, Sigma T8877) in PBS for 15 min at 37 °C, protected from light. Sections were transferred to cold 4% PFA, and later imaged using × 0.8 magnification on a stereo zoom microscope (Nikon SMZ-445). To ensure the right orientation of the brain sections, a small nick was made on the contralateral ventral region. Injury and hemisphere volumes were measured with ImageJ using the freehand selection tool and measuring the area. Volume was calculated as area by thickness of the slice (1 mm). Injury volume was expressed as % of ipsilateral hemisphere. At least three biological replicates were used for each time point (24 h, 7 days) and condition (sham, PT), and slices at 0.7, 1.7, 2.7 and 3.7 mm posterior to Bregma were analyzed, the slice at 3.7 mm posterior to Bregma being the posterior limit of the infarct. To evaluate the edematous expansion in the Chrdl1 KO compared to the WT we analyzed the difference between the volume of the ipsilateral hemispheres and the contralateral hemispheres, and used calculations previously described^35^: ipsilateral hemispheric volume (%) = [(Σ(I_i_ − C_i_))/(ΣC_i_)]*100, where I_i_ is the area of the ipsilateral hemisphere, including core of the injury, of slice i and C_i_ is the area of the contralateral hemisphere of slice i.

### Cell death detection

WT and Chrdl1 KO male mice littermates were subjected to sham surgeries or PT and their brains were collected 24 h later after 4% PFA perfusion. Brains were sliced in 16 µm-thick section and collected on Superfrost Plus micro slides (VWR 48311-703). In situ cell death detection kit TMR red (Roche 12156792910) was used to detect cell death using TUNEL technology in layers 2/3 of the visual cortex in the core of the injury and the contralateral hemisphere. The manufacturer’s instructions were followed. Brain sections were fixed with 4% PFA for 20 min at room temperature. Then brain sections were washed with PBS for 30 min at room temperature and permeabilized with 0.1% Triton X-100, 0.1% sodium citrate for 2 min at 4 °C. Brain sections were washed twice with PBS and incubated with the TUNEL mix (label solution + enzyme terminal transferase) for 60 min at 37 °C in a humidified chamber. For the positive controls, brain sections were prior treated with recombinant DNase I for 10 min at room temperature. For the negative controls, brain sections were incubated with the mix minus the enzyme terminal transferase (Fig. S6C). Brain sections were rinsed in PBS three times before mounting them with SlowFade gold antifade mountant with DAPI (Thermo Fisher Scientific S36939) and coverslips 22 × 50 mm 1.5 thickness sealed with nail polish.

Images were acquired in an Axio Imager.Z2 microscope, using a 20x/0.8 NA objective, as 16bit z-stacks (total thickness 1.96 µm, step size 0.49 µm, 10% overlap), image size 1388 × 1040 (pixel size 0.323 × 0.323 µm). Field of view represents random areas within the core. The z-stacks were analyzed as 3D images using IMARIS software (version 9.7; RRID:SCR_007370). DAPI and the TUNEL signal were defined as spheres of average diameter of 7 µm, and quantify a cell as TUNEL positive when both spheres within 3.5 µm distance from each other’s center. Representative images are orthogonal projections of maximum intensity.

### Spine imaging and analysis

YFP-Chrdl1 KO and YFP-WT male mice were subjected to sham surgeries or PT and perfused 24 h or 7 days later to obtain fixed tissue (see Tissue collection and preparation section). Slices were collected from fixed brains in the cryostat at 60 µm thickness at coordinates corresponding with the core of the injury (3.28 mm posterior and 2.80 mm lateral to Bregma) and surroundings to be able to image the peri-infarct area. Slices were placed on Superfrost Plus micro slides (VWR 48311-703) and mounted using SlowFade gold antifade mountant with DAPI (Thermo Fisher Scientific S36939) and coverslips 22 × 50 mm 1.5 thickness sealed with nail polish. Peri-infarct region (ipsilateral) and contralateral regions were imaged using a Zeiss LSM 880 Airyscan FAST super-resolution microscope. Images were taken using the 63x/1.4 NA oil-immersion objective, as 16-bit images at 1648 × 1648 pixels (pixel size 0.04 × 0.04 µm) as z-stacks of 30 steps with a total thickness of 5.51 µm. Representative images are orthogonal projections. Figure 7a was taken using a Thunder Imager 3D Tissue Microsystem (Leica) at 10x/0.32 NA as 16-bit images of 54 tiles with 10% overlap, image size 16,508 × 11,155 pixels (pixel size 0.65 × 0.65 µm).

Images were analyzed using NeuronStudio software (version 0.9.92; RRID:SCR_013798). Measurements were from secondary dendrites of neurons in layer 5 extending into layers 2/3 of the visual cortex in the more ventral region of the peri-infarct area and the equivalent region in the contralateral hemisphere. At least 6 dendrites per mouse were analyzed. Spine classifier within the software was set as 1.1 µm head-to-neck ratio threshold, 2.5 µm height-to-width ratio threshold and mushroom head size of 0.35 µm or larger in order to classify spines as immature (stubby or thin), or mature (mushroom)^39^.

### Quantification and statistical analysis

Mice were randomly assigned to sham or PT groups, and the experimenter was blind to genotype and condition (sham or PT) at the time of analysis. Sample size for each experiment was based on previous studies in the literature. No animals were excluded from the study. GraphPad Prism (version 8; RRID:SCR_002798) was used to design graphs and statistical analysis.

Results from Figs. 6, 7b–d, Figs. S5 and S6b–d were analyzed using one-way ANOVA with Tukey’s post-hoc test. Paired T-test was used to compare differences in the % of cells expressing Chrdl1 and Slc1a3, Tubb3 or other between sham and PT animals from Fig. 2d–f, and unpaired T-test to compare Chrdl1 threshold area between sham and PT WT mice from Fig. 2g–i, and the edematous expansion difference between WT and Chrdl1 KO in Fig. 5c. All the other experiments were analyzed using two-way ANOVA with Sidak’s post-hoc test. Results are shown as mean ± standard error with dots representing averaged results from at least 3 brain sections of one mouse, and graphs show exact p-values. Full statistical calculations for each figure are presented in Table S1, with each figure and the associated supplementary figure (when applicable) presented on a separate tab.

## Supplementary Information

Supplementary Tables.

Supplementary Figures.

## Data Availability

All data generated or analysed during this study are included in this published article (and its Supplementary Information files).
