# Supplementary material for: Astrocyte-secreted chordin-like 1 regulates spine density after ischemic injury
Source: Sci Rep. 2022 Mar 9;12:4176. doi: 10.1038/s41598-022-08031-4 (PMC8907233; doi:10.1038/s41598-022-08031-4)
Supplement: Supplementary file 2 — Supplementary Figures. [file 41598_2022_8031_MOESM2_ESM.pdf]

Astrocyte-secreted chordin-like 1 regulates spine density after ischemic injury

Elena Blanco-Suarez and Nicola J Allen

Supplementary Information
There are seven supplemental figures and one supplemental table associated with this manuscript.

Supplementary Table 1. This is an Excel file, where each tab contains full statistical analysis for data presented in each figure (Figures 1-7 and associated supplements).

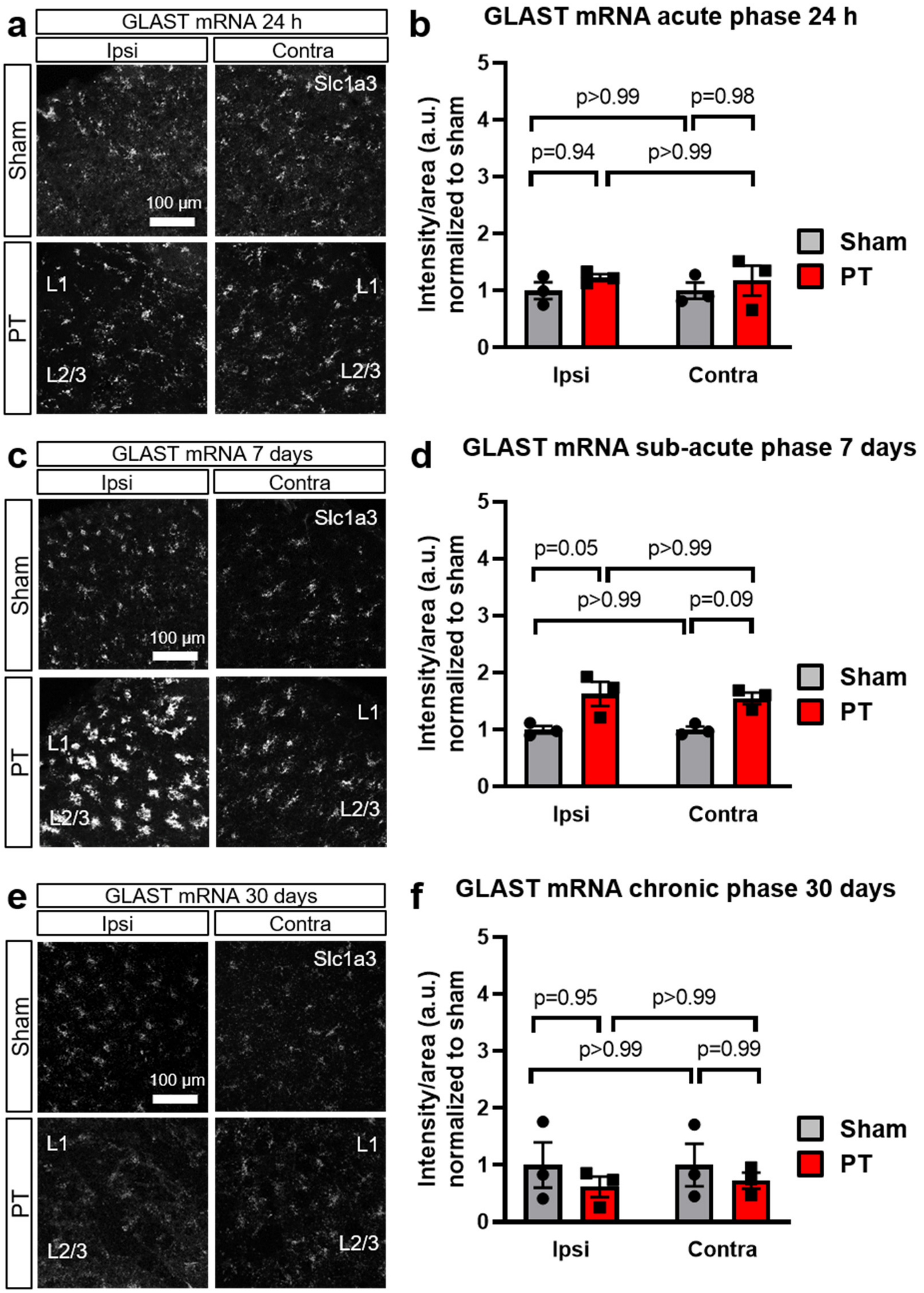

Figure S1, related to main Figure 1

Figure S1 (related to main Figure 1) GLAST expression increases in the peri—infarct area only during the sub-acute phase. (a) Representative images of FISH of GLAST (Slc1a3) in the peri-infarct area (ipsilateral hemisphere, ipsi) and in the contralateral hemisphere (contra) to the ischemic lesion of coronal sections 24 hours after PT or sham surgeries in WT mice. Images from layers 2/3 of the visual cortex. (b) Quantification of fluorescence intensity per unit area normalized to sham. Sham N=3, PT N=3 mice. (c, e) Same as a, 7 days and 30 days after PT or sham surgeries, respectively. (d, f) Same as b, 7 days and 30 days after PT or sham surgeries, respectively. Sham N=3, PT N=3 mice both timepoints. Statistics by two-way ANOVA. Scale bar 100 µm.

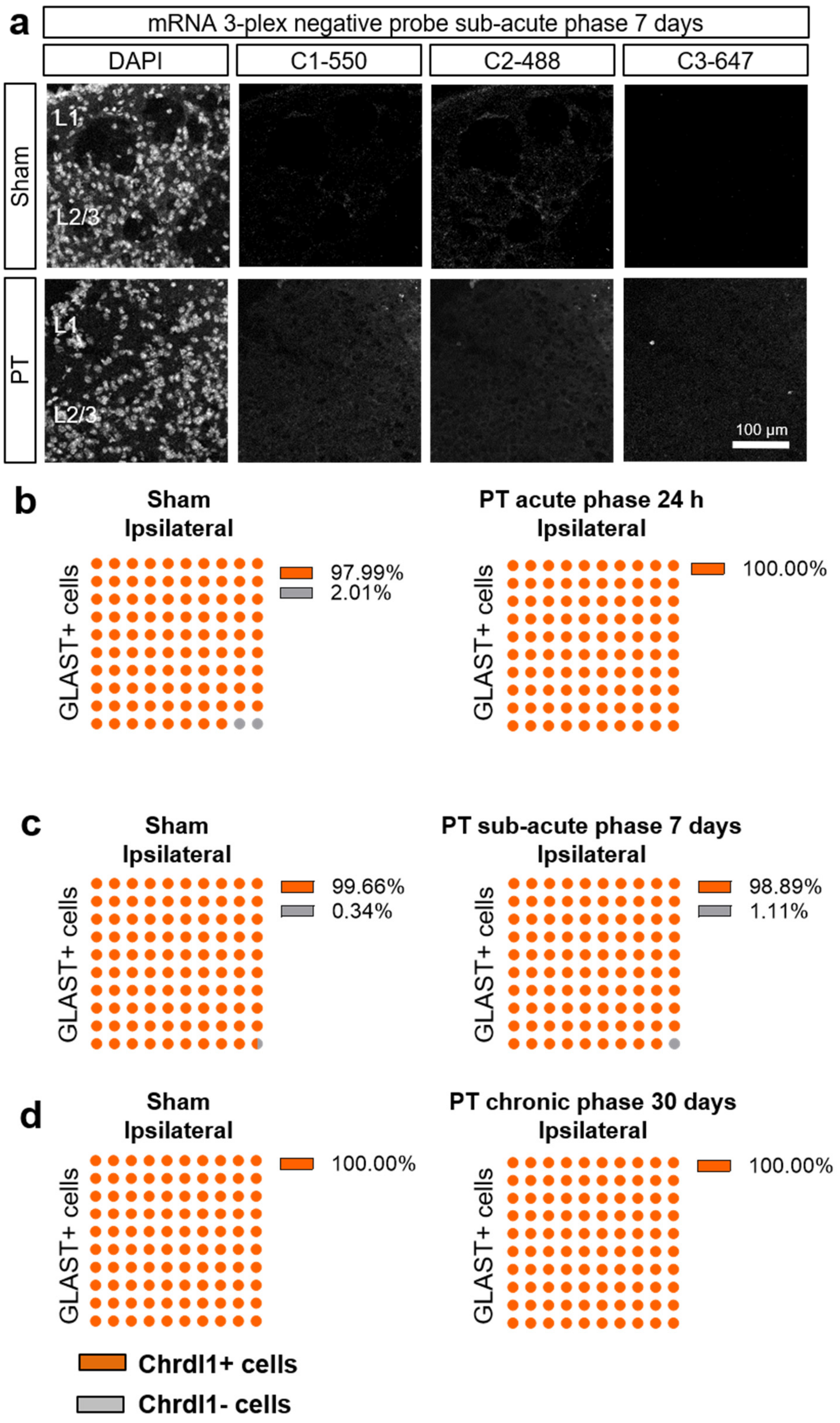

Figure S2, related to main Figure 2

Figure S2 (related to main Figure 2) Astrocytes are the main cells expressing Chrdl1 in the peri-infarct area. (a) Images of the peri-infarct area of WT mice 7 days after sham surgeries or PT with FISH negative control probe from each channel (C1, C2 and C3 and their respective excitation). Signal detected was minimal. Scale bar 100 µm. (b, c, d) Graphic representation of % of GLAST(Slc1a3)+ cells that were found to express Chrdl1 signal (in orange) or not (gray), across all samples (Sham N=3, PT N=3 mice per time point).

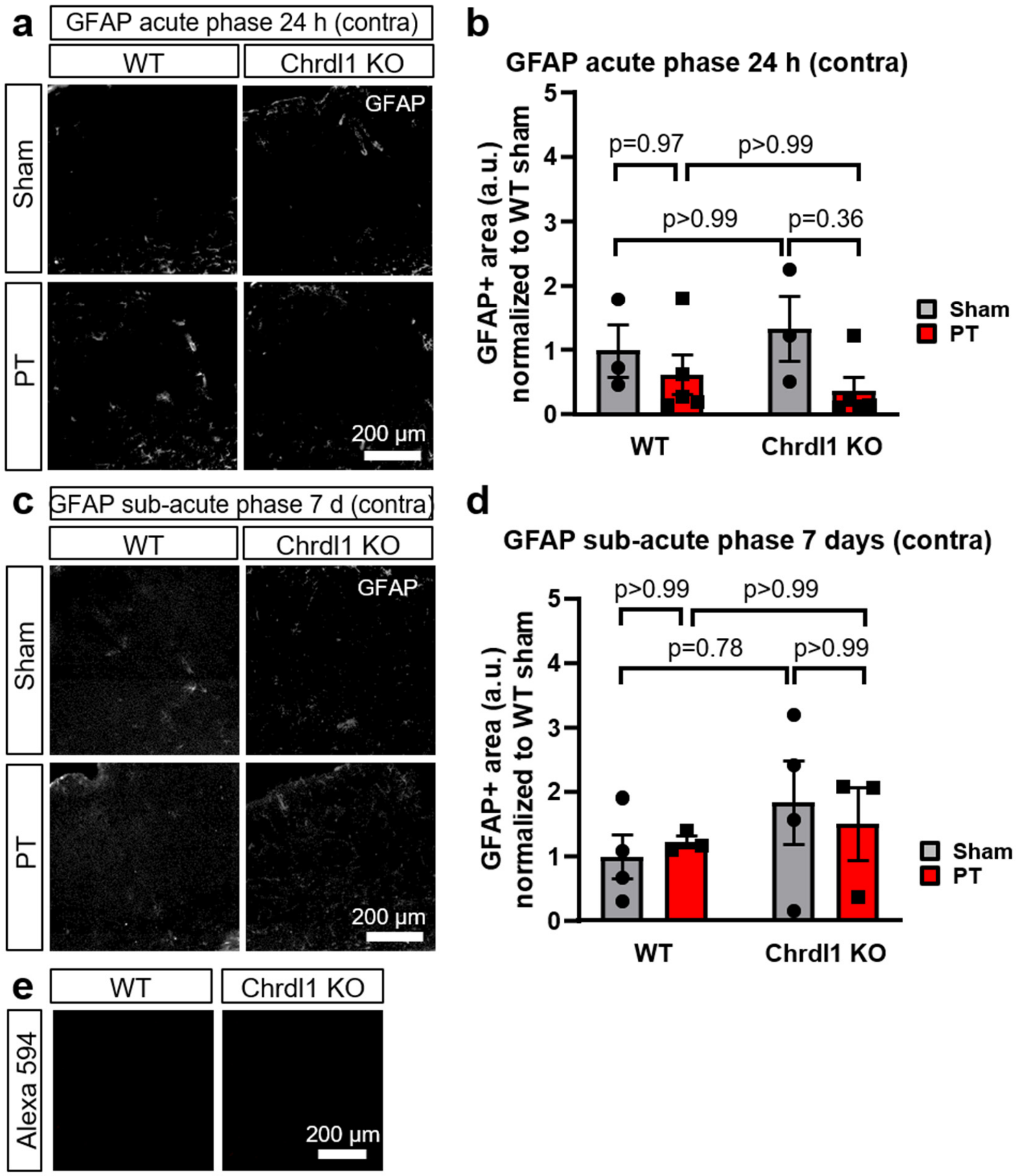

Figure S3, related to main Figure 3

Figure S3 (related to main Figure 3) Reactive astrogliosis is not impaired in Chrdl1 KO mice after PT injury. (a) Representative images of the ROI in the homologous contralateral hemisphere to the PT lesion of WT and Chrdl1 KO mice 24 hours after PT or sham surgeries immunostained for GFAP (b) Quantification of GFAP+ stained area normalized to WT sham. Statistics by two-way ANOVA. WT sham N=3, WT PT N=5, Chrdl1 KO sham N=3 and Chrdl1 KO PT N=5 mice. (c, d) Same as a and b 7 days after PT or sham surgeries. WT sham N=4, WT PT N=3, Chrdl1 KO sham N=4 and Chrdl1 KO PT N=3 mice. Scale bar 200 μm. (e) Negative controls of the peri-infarct area of a WT and a Chrdl1 KO mouse 7 days after PT. Primary antibody was omitted from the immunostaining. Only secondary antibody (anti-rabbit Alexa 594) was used. No signal was detected. Scale bar 200 μm.

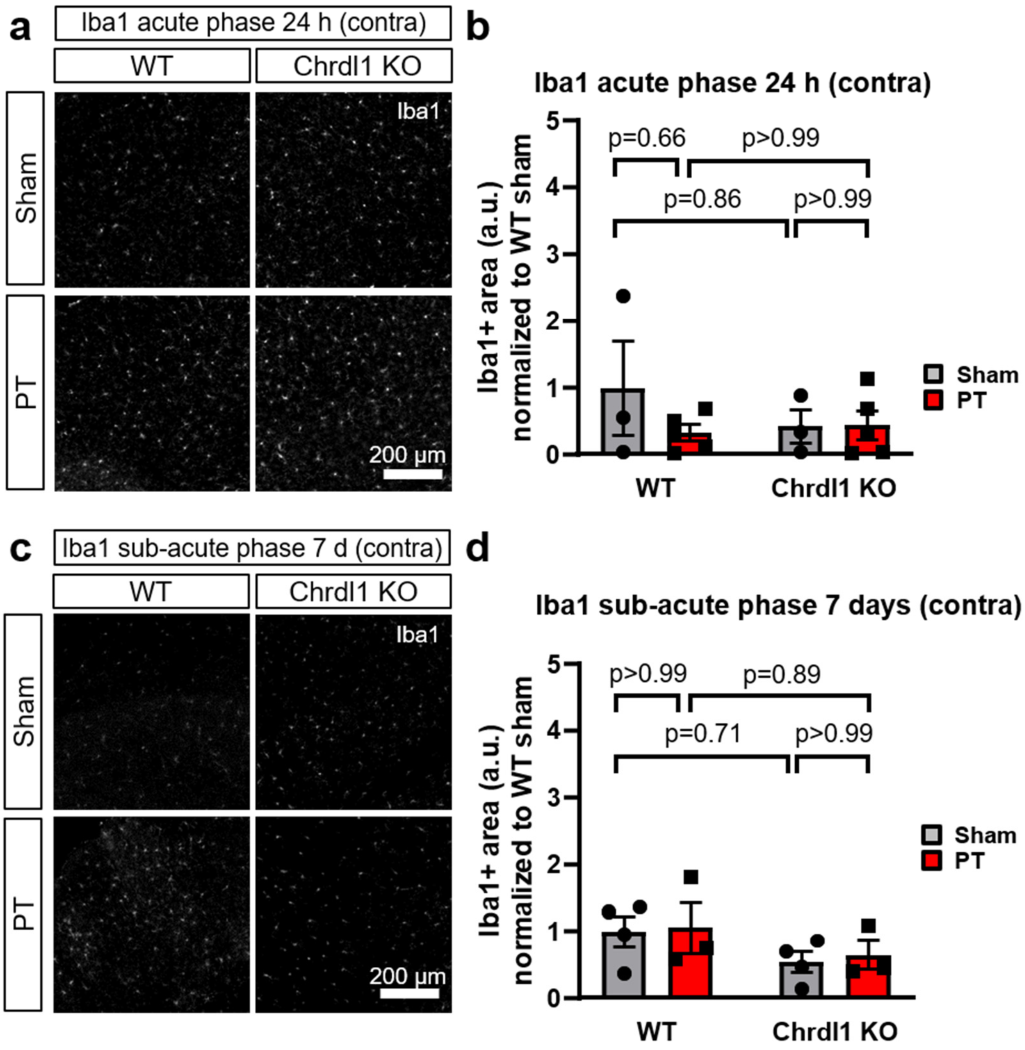

Figure S4, related to main Figure 4

Figure S4 (related to main Figure 4) Absence of Chrdl1 does not affect the microglia response after PT injury. (a) Representative images showing Iba1 immunostaining of the ROI in the homologous contralateral hemisphere to the injury of WT and Chrdl1 KO mice 24 hours after PT or sham surgeries. (b) Quantification of Iba1+ stained area normalized to WT sham. Statistics by two-way ANOVA. WT sham N=3, WT PT N=5, Chrdl1 KO sham N=3 and Chrdl1 KO PT N=5 mice. (c, d) Same as a and b, 7 days after PT or sham surgeries. WT sham N=4, WT PT N=3, Chrdl1 KO sham N=4 and Chrdl1 KO PT N=3 mice. Scale bar 200 µm.

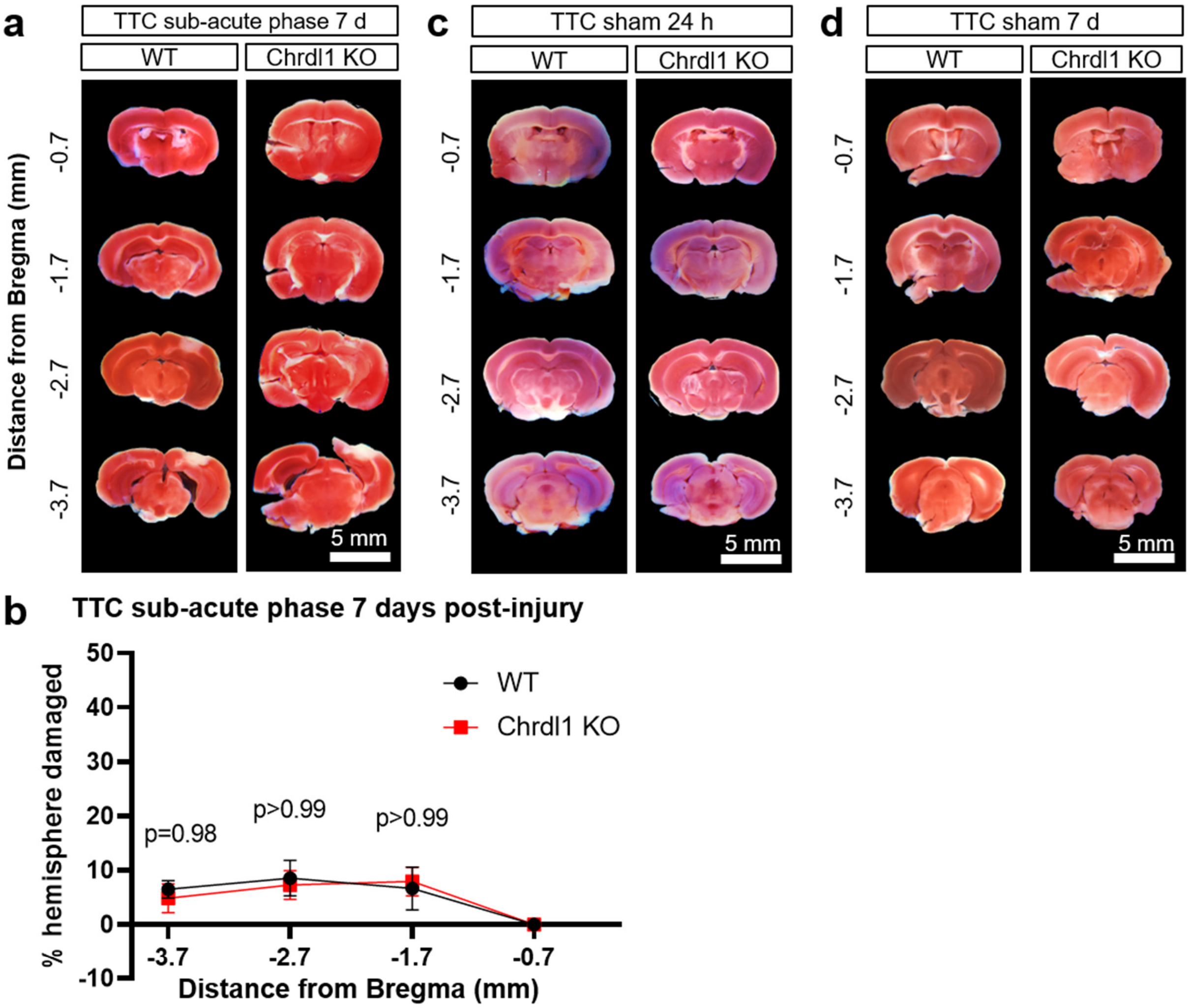

Figure S5, related to main Figure 5

Figure S5 (related to main Figure 5) Absence of Chrdl1 does not affect PT injury volume. (a) Representative images of 1 mm-thick coronal sections of WT and Chrdl1 KO mouse brains 7 days after PT stained with TTC, corresponding to 0.7, 1.7, 2.7 and 3.7 mm posterior from Bregma. The dead tissue (brain regions infarcted) is not stained by TTC and appears white, whereas the rest of the tissue turns a shade of red. Scale bar 5 mm. (b) Quantification of the volume of the injury as percentage of the total volume of the ipsilateral hemisphere. WT N=4, Chrdl1 KO N=6. Statistics by two-way repeated measures ANOVA. (c, d) Representative images of the same experiment but 24 hours or 7 days after sham surgeries. No infarct tissue is present. 24 hours post-sham: WT N=4, Chrdl1 KO N= 3; 7 days post-sham: WT N=4, Chrdl1 KO N=4 mice. Scale bar 5 mm.

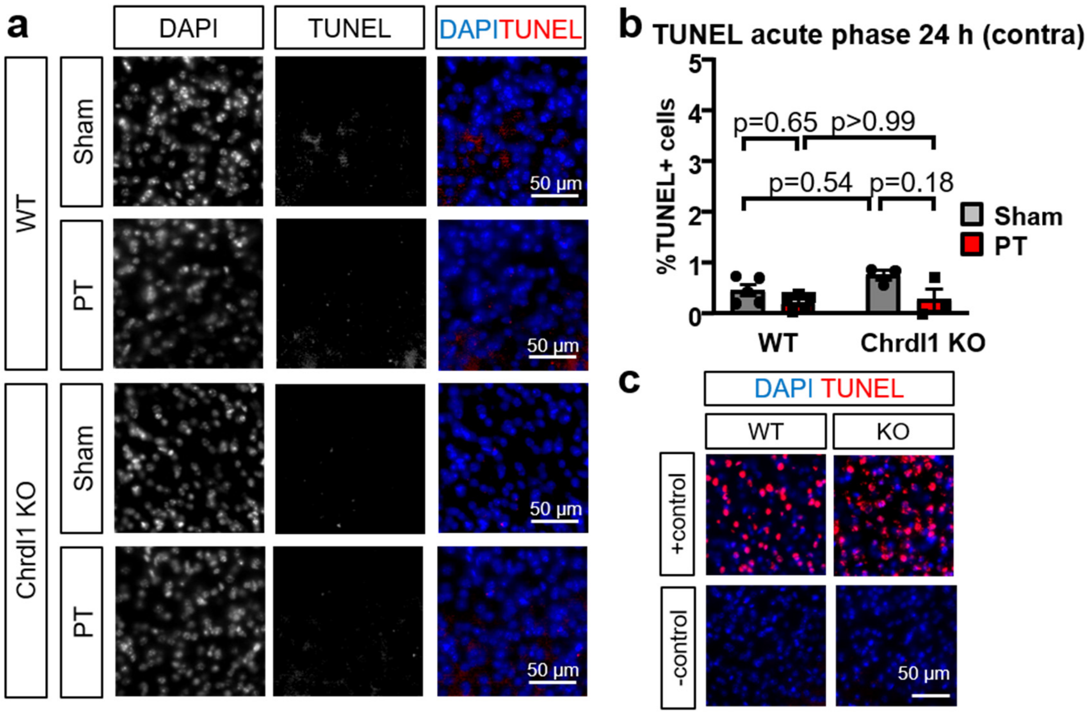

Figure S6, related to main Figure 6

Figure S6 (related to main Figure 6) Cell death is reduced in Chrdl1 KO mice after PT injury. (a) Example images of TUNEL staining of WT and Chrdl1 KO contralateral (contra) area, homologous to the core of the injury, in layers 2/3 of the visual cortex 24 hours after surgery. Scale bar 50 µm (b) Quantification of the % of cells stained with TUNEL. WT sham N=5, WT PT N=5, Chrdl1 KO sham N=3, Chrdl1 KO PT N=3 mice. Statistics by two-way ANOVA. (c) Example images of positive and negative controls for TUNEL staining, in homologous area to core of the injury of layers 2/3 of the visual cortex where analysis was made. Scale bar 50 µm.

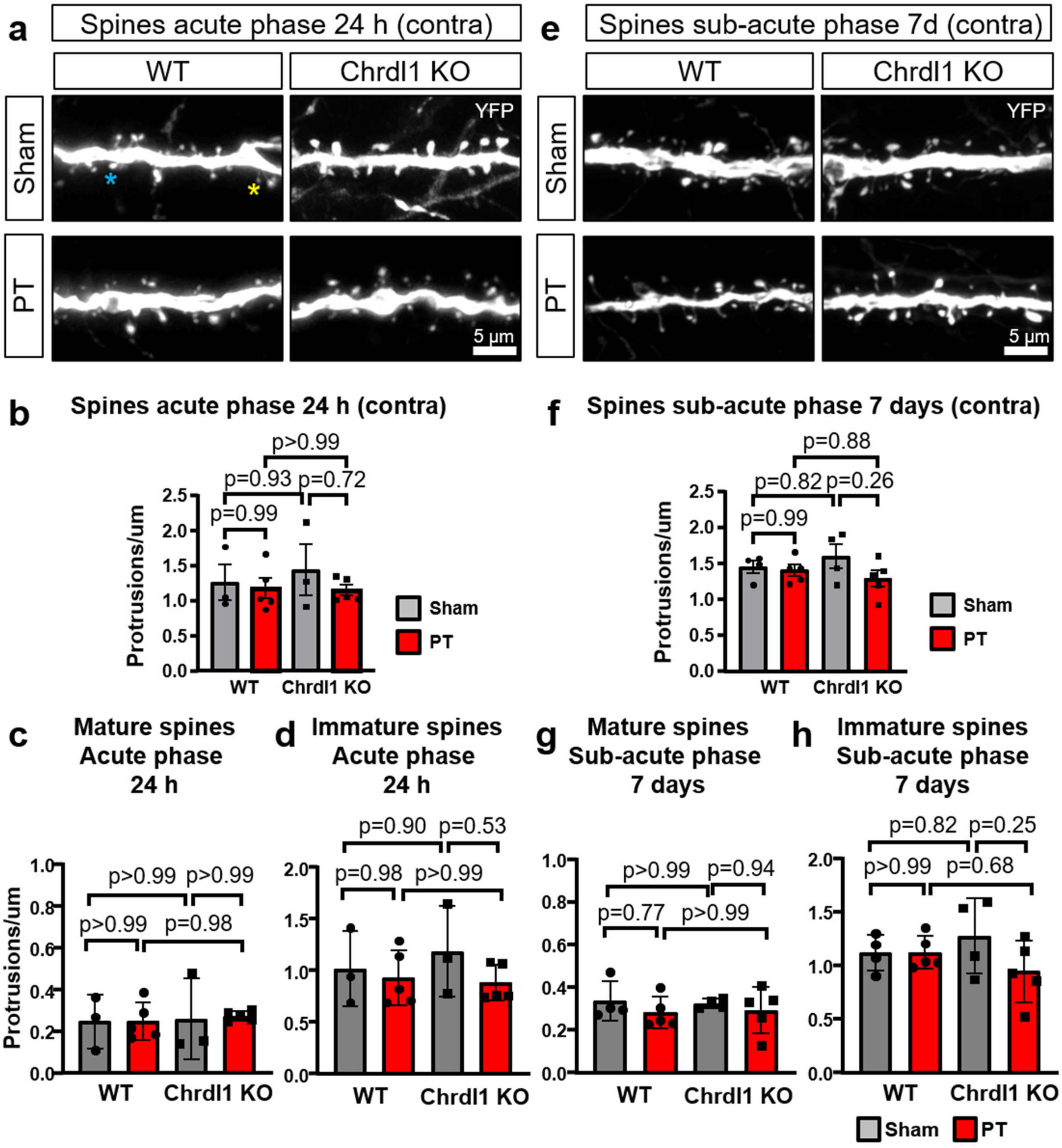

Figure S7, related to main Figure 7

Figure S7 (related to main Figure 7) Absence of Chrdl1 prevents spine loss in the peri-infarct area. (a) Representative images of secondary dendrites of layer 5 neurons expressing YFP in WT or Chrdl1 KO mice, in layers 2/3 of the visual cortex 24 hours after PT or sham surgeries in the contralateral area to the injury. Blue star indicates an example of a spine of mature morphology, and the yellow star indicates an example of a spine of immature morphology. Scale bar 5 µm. (b) Quantification in the contralateral area of number of spines per µm on dendrites in layers 2/3 of the visual cortex of animals 24 hours after PT or sham surgery. Statistics by one-way ANOVA. WT sham N=3, WT PT N=5, Chrdl1 KO sham N=3, Chrdl1 KO PT N=5 mice. (c) Quantification in the contralateral area of spines with mature morphology on dendrites in layers 2/3 of the visual cortex of animals 24 hours after PT or sham surgery. Statistics by one-way ANOVA. WT sham N=3, WT PT N=5, Chrdl1 KO sham N=3, Chrdl1 KO PT N=5 mice. (d) Quantification in the contralateral area of spines with immature morphology on dendrites in layers 2/3 of the visual cortex of animals 24 hours after PT or sham surgery. Statistics by one-way ANOVA. WT sham N=3, WT PT N=5, Chrdl1 KO sham N=3, Chrdl1 KO PT N=5 mice. (e) Same as a, 7 days after PT or sham surgeries in the contralateral area to the injury. Scale bar 5 µm. (f) Same as b, 7 days after PT or sham surgeries in the contralateral area to the injury. Statistics by one-way ANOVA. WT sham N=4, WT PT N=5, Chrdl1 KO sham N=4, Chrdl1 KO PT N=5 mice. (g) Same as c, 7 days after PT or sham surgery. Statistics by one-way ANOVA. WT sham N=4, WT PT N=5, Chrdl1 KO sham N=4, Chrdl1 KO PT N=5 mice. (h) Same as d, 7 days after PT or sham surgery. Statistics by one-way ANOVA. WT sham N=4, WT PT N=5, Chrdl1 KO sham N=4, Chrdl1 KO PT N=5 mice.
